# Profiling Secreted miRNA Biomarkers of Chemical-Induced Neurodegeneration in Human iPSC-Derived Neurons

**DOI:** 10.1093/toxsci/kfac011

**Published:** 2022-02-04

**Authors:** Dahea You, Jennifer D Cohen, Olga Pustovalova, Lauren Lewis, Lei Shen

**Affiliations:** Drug Safety Research & Evaluation, Takeda Development Center Americas, Inc., San Diego, California 92121-1964, USA; Drug Safety Research & Evaluation, Takeda Development Center Americas, Inc., San Diego, California 92121-1964, USA; Clarivate, Philadelphia, Pennsylvania 19130, USA; Drug Safety Research & Evaluation, Takeda Development Center Americas, Inc., Cambridge, Massachusetts 02139, USA; Data Science Institute, Takeda Development Center Americas, Inc., Cambridge, Massachusetts 02139, USA

**Keywords:** human iPSC-neurons, neurodegeneration, neurotoxicity, high-content imaging, TGF-β, pathway, miRNA biomarkers

## Abstract

Elucidation of predictive fluidic biochemical markers to detect and monitor chemical-induced neurodegeneration has been a major challenge due to a lack of understanding of molecular mechanisms driving altered neuronal morphology and function, as well as poor sensitivity in methods to quantify low-level biomarkers in bodily fluids. Here, we evaluated 5 neurotoxicants (acetaminophen [negative control], bisindolylmaleimide-1, colchicine, doxorubicin, paclitaxel, and rotenone) in human-induced pluripotent stem cell-derived neurons to profile secreted microRNAs (miRNAs) at early and late stages of decline in neuronal cell morphology and viability. Based on evaluation of these morphological (neurite outgrowth parameters) and viability (adenosine triphosphate) changes, 2 concentrations of each chemical were selected for analysis in a human 754 miRNA panel: a low concentration with no/minimal effect on cell viability but a significant decrease in neurite outgrowth, and a high concentration with a significant decrease in both endpoints. A total of 39 miRNAs demonstrated significant changes (fold-change ≥ 1.5 or ≤ 0.67, *p* value < .01) with at least 1 exposure. Further analyses of targets modulated by these miRNAs revealed 38 key messenger RNA targets with roles in neurological dysfunctions, and identified transforming growth factor-beta (TGF-β) signaling as a commonly enriched pathway. Of the 39 miRNAs, 5 miRNAs, 3 downregulated (miR-20a, miR-30b, and miR-30d) and 3 upregulated (miR-1243 and miR-1305), correlated well with morphological changes induced by multiple neurotoxicants and were notable based on their relationship to various neurodegenerative conditions and/or key pathways, such as TGF-β signaling. These datasets reveal miRNA candidates that warrant further evaluation as potential safety biomarkers of chemical-induced neurodegeneration.

Neurotoxicity accounts for approximately a quarter of adverse effects observed in clinical trials and is the third leading cause of safety-related drug attrition (Cook *et al.*, 2014; Monticello *et al.*, 2017; Walker *et al.*, 2018). Low concordance of nonclinical assays to predict the most common nervous system-related adverse events in clinical trials has been cited as partially causative of the high clinical attrition rates from neurotoxicity (Mead *et al.*, 2016; Ross, 2000). Neurotoxic findings may be missed in nonclinical studies due to: (1) a limited number of sample timepoints for histopathology, (2) a limited number of observations for the modified Irwin (FOB/Irwin) neurological behavioral assay, (3) a lack of translation of neurological findings in nonclinical species to humans, (4) infrequent use of electroencephalogram to identify electrophysiological disturbances and, (5) infrequent use of special stains for neuro-histopathology (eg, Fluoro-Jade, Alcian blue, Toluene blue, IBA1, GFAP) to identify discreet lesions (Mathiasen and Moser, 2018; Moscardo *et al.*, 2007; Nagayama, 2015; Switzer, 2011). Therefore, elucidation of fluidic biomarkers to detect chemical-induced neurotoxicity may improve identification of neurotoxic risk in nonclinical studies and reduce neurotoxicity-related clinical attrition.

Currently, no fluid-based biomarker has been validated for diagnosis or prognosis of nonclinical or clinical chemical-induced neurodegeneration, but scientific literature is rapidly expanding in this area (Imam *et al.*, 2018; Kim *et al.*, 2020; Meregalli *et al.*, 2020; Walker *et al.*, 2018). In recent exploratory rodent or clinical studies with chemotherapeutics, increases in serum neurofilament light (NfL) protein levels have correlated well to severity of peripheral neuropathy. In rats treated with chemotherapeutics cisplatin and paclitaxel (PTX) for 4 weeks, temporal increases in serum NfL levels correlated to severity of axonal damage in peripheral nerve tissues, decreases in nerve fiber density measurements, and/or reductions in sensory nerve action potential amplitude (Meregalli *et al.*, 2020). In a clinical trial with oxaliplatin, increases in serum NfL following 3 or 6 months of treatment correlated with severity of oxaliplatin-induced peripheral neuropathy in patients (Kim *et al.*, 2020). Although it is encouraging that NfL is gaining traction as a safety biomarker for neurodegeneration, continued exploration of diverse markers will be beneficial to understanding the mechanisms of chemical-induced neurodegeneration and to building an arsenal of safety biomarkers for translational monitoring from nonclinical species to humans.

Although typical fluid biomarkers are protein or lipids, microRNAs (miRNAs) are rapidly emerging as impactful targets for fluid-based biomarker discovery due to their stability in the blood and conservation across species (Di Pietro *et al.*, 2021; Imam *et al.*, 2018). miRNAs are small noncoding RNA, approximately 18–25 nucleotides long, which are posttranscriptionally transcribed from highly conserved intra- or intergenic regions of the genome that regulate gene expression (Ambros *et al.*, 2003; Bartel, 2009; Denk *et al.*, 2015). Mature miRNA regulates protein synthesis by loading into RNA-induced silencing complex to target messenger RNA (mRNA) using Watson-Crick base pairing leading to either cleavage or destabilization and subsequent degradation or translational repression of the mRNA (Kim, 2005a,b; Pasquinelli, 2012). Approximately 2585 unique human miRNAs have been discovered. In an OpenArray profiling of 1178 miRNAs in cerebrospinal fluid (CSF) from Alzheimer’s disease (AD) patients, 441 miRNAs were determined to be expressed, signifying that at least 37% of those miRNAs are expressed in the brain (Denk *et al.*, 2015). miRNAs hold great potential as peripheral biomarkers for neurotoxicity since they play a vital role in neuronal cell development, proliferation, differentiation, function, homeostasis, maintenance, and apoptosis (Denk *et al.*, 2015; Kaur *et al.*, 2012; Sun and Shi, 2015).

In this study, we aimed to identify secreted miRNA biomarkers and their associated pathways that were similarly dysregulated by classic neurotoxicants with diverse biological mechanisms of toxicity. Human-induced pluripotent stem cells (hiPSC)-derived gamma-aminobutyric acid (GABA) neurons, previously found to be sensitive to chemical treatments (Cohen and Tanaka, 2018), were exposed to neurotoxicants (acetaminophen [ACET; negative control], bisindolylmaleimide-1 [BIS], colchicine [COL], doxorubicin [DOX], PTX, and rotenone [ROT]). The complex effects of these toxicants on neuronal cell injury were assessed using an integrative approach across multiple endpoints, including: (1) cell viability, measured by adenosine triphosphate (ATP) analysis; (2) neuronal morphological abnormalities, quantified by high-content imaging analysis; (3) dysregulation of miRNA expression using a human miRNA panel; and (4) elucidation of commonly enriched neurodegenerative pathways. We identified 39 secreted miRNAs that were significantly modulated in hiPSC-derived neurons upon chemical-induced neurodegeneration. Pathway analysis identified several processes associated with the 39 miRNAs, with transforming growth factor-beta (TGF-β) signaling being the pathway most commonly enriched. Of the 39 miRNAs, 5 (miR-20a, -30b, -30d, -1234, and -1305) were most notable based on their significant dysregulation by multiple chemicals and their involvement in pathways related to neurological dysfunction, including TGF-β signaling pathways. Our study demonstrated that these 5 miRNAs are potential noninvasive biomarkers for chemical-induced neurodegeneration.

## MATERIALS AND METHODS

###  

####  

##### Cell culture

hiPSC-derived cortical GABAergic iCell Neurons (Fujifilm Cellular Dynamics International, Madison, Wisconsin), derived from a healthy, Caucasian, female donor over the age of 18, with a normal genetic background (https://www.fujifilmcdi.com/icell-gabaneurons-01434-ggbn01434, accessed on January, 2022) were cultured according to the manufacturer’s protocol. Cells were plated at a density of 10 000 live cells/well on BioCoat Poly-D-Lysine 96-well tissue culture plates (BD Biosciences; San Diego, California) coated with 3.3 µg/ml laminin. At 2 h postplating, cells were treated with chemicals (ACET, negative control; BIS; COL; DOX; PTX; ROT) at 0.1, 1, 10, 30, and 100 µM in 0.1% dimethyl sulfoxide (DMSO, purchased from Sigma-Aldrich, Calbiochem, or Wako) with 3 technical replicates per chemical concentration per plate. Control wells were treated with 0.1% DMSO. Chemical and control wells were incubated at 37°C for 24 h prior to fixation or ATP analysis. The optimal timing for treating cells at 2 h postplating and the 24 h chemical treatment duration were determined based on time course results from Cohen and Tanaka (2018).

##### ATP analysis

ATP was analyzed using the CellTiter-Glo Luminescent Cell Viability Assay (Promega, Madison, Wisconsin) according to manufacturer’s recommendations, and luminescent signal was quantified using an Envision 2104 Multilabel Reader (Perkin Elmer, Foster City, California). Data were analyzed using Dunnett’s test, with the level of significance set at *p* ≤ .05.

##### Immunocytochemistry and automated image analysis

Cultures were fixed with 8% paraformaldyde (Alfa Aesar, Heysham, UK) in Dulbecco’s phosphate-buffered saline (D-PBS) without Ca^2+^ and Mg^+^ (Wako, Osaka, Japan) for 1 h, and permeabilized with 0.3% triton X100 (Wako) in blocking buffer containing D-PBS solution of 10% donkey serum (Millipore, Billerica, Massachusetts) and 1% bovine serum albumin (Jackson Immuno Research Laboratories, Inc., West Grove, Pennsylvania). Neurons were labeled with an anti-BIII-tubulin primary antibody (G712A, Promega) at 1:200 dilution in blocking buffer overnight at 4°C, washed with D-PBS, labeled with secondary antibody Alexa Fluor 488-goat-antimouse (Invitrogen, Carlsbad, California) for 1 h at room temperature in blocking buffer, washed with D-PBS, and incubated with SlowFade Gold Antifade Mountant with DAPI (Invitrogen) prior to automated image analysis.

##### Automated image analysis

Automated image acquisition was performed on an In Cell Analyzer 6000 high-content imaging system (GE Healthcare UK Ltd., Buckinghamshire, UK) and image analysis of neuronal morphology parameters was quantified using the Neuronal Morphology Algorithm on the GE IN Cell Developer Toolbox 1.9.2 (GE Healthcare) as previously described (Cohen and Tanaka, 2018). Images were acquired with a 20× objective, across 16 image-fields per well, for all groups. Using the Neuronal Morphology Algorithm we quantified morphological changes of neurons, including neurite length and neurite count, and cell density by quantifying neurons per field, following chemical treatments. For statistical analysis of morphological endpoints, 2 biological replicates for each set of experiments (*n* = 3 wells/concentration/chemical/plate), with independent cultures, were combined for a final group size of *n* = 6 wells/concentration of chemical. Data were first normalized within each separate plate, and then pooled across biological replicates to obtain composite profiles for each chemical concentration. Combined data were analyzed using Dunnett’s test, with the level of significance set at *p* < .05, to determine the lowest effect concentration of each chemical with a significantly different morphological response from control. Neurite count and neurite length data were assessed for their correlation with the ATP changes from the cell viability assay described earlier. For this, we performed Pearson’s correlation analysis using R Studio Pro Version 1.2.5033-1. Correlation coefficient and corresponding *p* value were determined for each comparison.

##### RNA extraction from neurotoxicant-treated neuronal cell culture supernatant

Culture supernatant from 3 technical replicates per concentration per plate was pooled into a single biological replicate, and centrifuged at 2000 × g for 10 min, followed by 10 000 × g for 20 min at 4°C to remove cells and cell debris. Small RNA-containing total RNA was isolated using miRNeasy Micro Kit (Qiagen; Hilden, Germany) according to the manufacturer’s instructions. Briefly, 0.12 ml of culture supernatant was mixed with 0.6 ml of QIAzol Lysis reagent by vortexing. After adding 5 µl of 500 pM Ath-miR-159a (synthesized by Hokkaido System Science, Sapporo, Japan), the mixture was incubated for 5 min at room temperature. Next, 120 µl of chloroform (Wako) was added followed by vigorous mixing by hand for 15 s. After incubation for 2 min at room temperature, phase separation was performed by centrifugation at 12 000 × g for 15 min at 4°C. From the upper aqueous phase, 0.42 ml was transferred to a new tube and then 1.5-fold volume of 99.5% ethanol was added. The mixture was passed through an RNeasy MinElute Spin Column by vacuum. After washing of the cartridge once with 700 µl of Buffer RWT and twice with 500 µl of Buffer RPE by vacuum, elution of total RNA was performed with 14 µl of RNase-free water and centrifugation (8000 × g, 1 min, RT). The RNA quality and yield were analyzed using an Agilent 2100 Bioanalyzer with RNA6000 Pico Kit (Agilent Technologies, Santa Clara, California). A centrifugal vacuum evaporator was used to concentrate the eluent and the volume was adjusted up to 7 µl with nuclease-free water.

##### TaqMan array quantitative analysis of miRNA

For each chemical treatment and control, 3 biological replicates were performed for miRNA quantification using TaqMan Array Human MicroRNA A + B Cards Set v3.0, which contains a primer set for 754 human miRNA sequences and was used according to the manufacturer’s instructions (Thermo Fisher Scientific, Waltham, Massachusetts). Synthesis of cDNA was carried out from 6 µl of RNA solution (3 µl for each primer pool) with Megaplex RT primers (pool A v.2.1 and pool B v.3.0) and TaqManTM MicroRNA Reverse Transcription Kit (Thermo Fisher Scientific). cDNA was amplified by use of Megaplex PreAmp primers pool A v.2.1 and pool B v.3.0 and TaqManTM PreAmp Master Mix. The amplified cDNA was mixed with TaqMan Universal PCR Master Mix (No AmpErase UNG). The obtained sample was loaded to TaqMan Array Human MicroRNA A + B Cards subjected to quantitative PCR analysis by use of 7900HT Fast Real-Time PCR system (Thermo Fisher Scientific). Thermal cycling condition was 2 min at 50.0°C, 10 min at 94.5°C, 40 cycles at 97.0°C for 30 s, and at 59.7°C for 1 min.

##### miRNA data analysis

Threshold cycle (*C*_t_) values were determined using RQ Manager 1.2 software (Thermo Fisher Scientific), setting the normalized fluorescence threshold to 0.2. Raw *C*_t_ values ≥ 35 were treated as below the detection limit and were assigned a raw *C*_t_ value of 35. Raw *C*_t_ values were further normalized within each plate (containing all samples belonging to a compound treatment) using the “mean-centered” approach: the mean of raw *C*_t_ values of each plate was calculated without values ≥ 35 (ie, below detection limit), and the normalized expression values were obtained by subtracting the raw *C*_t_ values from the mean *C*_t_ value within the same plate. As a result, the normalized expression value has the opposite trend of the meaning of a *C*_t_ value: the higher the expression, the higher the normalized expression value. After normalization, paired *t* tests comparing normalized expression values for the treatment versus control were completed for all miRNAs. We also performed multiple-testing correction by calculating the Storey’s *q* values, and adjusted *p* values using the Benjamini-Hochberg approach (Storey, 2002; Storey and Tibshirani, 2003), to further assess statistical significance. To select relevant miRNA candidates for follow-up analyses using ontology of biological pathways, we introduced an additional constraint on strength of effect size (fold-change ≥ 1.5 or ≤ 0.67), and applied an unadjusted *p* value < .01. These results were reported in Supplementary Table 1. The initial risk for random chance using the unadjusted *p* value was mitigated by the biological connection with the additional enrichment analysis, providing a level of confidence that the signal was likely biological and not due to chance. Based on these thresholds we identified 41 significant miRNAs. We used Thermo Fisher Scientific’s annotation file to annotate miRNA nomenclature with Assay IDs and miRBase IDs (megaplex-pools-array-card-content.xlsx annotation file downloaded from the product section of Thermo Fisher Scientific website [www.thermofisher.com], accessed on February 18, 2017). We annotated miRNAs with mirBase Accession IDs (miRbase v21), MetaBase Network Object IDs, and with associated Entrez Gene IDs (v.6, 2017). Thus, we were able to remap original Thermo Fisher Scientific miRNA names to Entrez Gene IDs and MetaBase Network Objects for enrichment and network-based analysis. We excluded 2 miRNA IDs from the analysis, which did not map to Entrez Gene IDs. These miRNA names either mapped to discontinued Entrez Gene IDs or did not map to human miRNA per Thermo Fisher annotation file. Ultimately, 39 significant miRNAs successfully mapped to Entrez Gene IDs and were used in subsequent pathway analyses (Supplementary Table 1).

##### Pathway enrichment analysis

The 39 significant miRNAs, identified by methods described above, were clustered into 4 groups based on fold-change values, using hierarchical clustering with average linkage and Euclidian distance. Clusters were identified through dynamic tree cut method using the R package (dynamicTreeCut 1.63-1). We utilized 2 different miRNA pathway analysis tools, mirPath v.3 and Clarivate Pathway Maps, to determine neurodegeneration-related molecular pathways regulated by differentially expressed miRNAs.

Each of the 4 clusters was analyzed by mirPath v.3, which is a web-based tool enabling target prediction and pathway enrichment for miRNAs of interest (Vlachos et al., 2015; accessible at: http://snf-515788.vm.okeanos.grnet.gr/, accessed 4 March 2021). Significant miRNAs from each cluster (input lists available in Supplementary Table 2) were uploaded into this software. The settings for the analysis were as follows: Kyoto Encyclopedia or Genes and Genomes (KEGG) analysis with human for species and microT-CDS (v5.0) for the database. Pathway Union approach was utilized to identify all significant pathways within KEGG that involve gene targets of at least 1 miRNA in the input list. The *p* value for each pathway was calculated using Fisher’s meta-analysis method, which reflects the probability of the pathway’s enrichment with the gene targets of at least 1 selected miRNA in the input list. Pathways with *p* values < .05 were considered significant. The list of significant pathways for each cluster of significant miRNAs was compared and the pathways that were commonly associated with all 4 clusters were identified.

The second approach utilized a proprietary Clarivate Pathway Maps collection, which includes more than 1500 maps. Each map encompasses 3–5 signaling pathways in normal or pathological condition, curated by Clarivate scientists and guided by experts in a particular disease area during the map creation. The collection includes maps specific to central nervous system diseases, including Huntington’s disease, multiple sclerosis (MS), and depression, which were relevant for the present analysis of neuronal cell toxic pathology.

As the role of miRNA in regulation of specific signaling cascades is still poorly understood, miRNA signaling is sparsely represented in the collection of pathway maps. This misrepresentation may lead to incorrect interpretation of enrichment analysis in miRNA. To avoid this bias, instead of direct enrichment of miRNAs in the pathway maps, we developed 2 methods:

1. *Method 1: Enrichment of miRNA targets in Clarivate pathway maps instead of miRNAs themselves*. Genes that are not known to be associated with any known miRNA were excluded from the pathway maps ontology. Targets of miRNAs were identified in Clarivate proprietary molecular interaction network with over 1 532 340 interactions manually curated by Clarivate scientists from published experimental papers. This method identified signaling cascades that were most affected by changes in the input miRNA profile. The disadvantage of the approach was potentially disproportionate contribution of the unbalanced number of targets associated with input miRNAs.
2. *Method 2: Enrichment of miRNAs in the pathway maps transformed into sets of miRNAs, which have at least* *1* *target gene present in the map*. The method is not affected by the bias introduced by the unbalanced number of miRNA targets, as described earlier. But, it may unfairly boost contribution of miRNAs with small number of targets.

Both enrichment methods described above estimate enrichment using hypergeometric distribution significance testing. As both methods are complementary and compensate each other’s biases, we took advantage of both methods and selected pathways identified as significant in both approaches with a Benjamini-Hochberg adjusted *p* value < .01. The selected terms were further ranked by cumulative *p* value calculated by Fisher’s method across both enrichment methods. For visualizations in this analysis, we further limited the results to the top 20 pathway maps with *p* value < .001 in the results from both methods, and clustered the pathways using hierarchical clustering with average linkage and Euclidian distance. Clusters were identified through a dynamic tree cut method implemented in R package dynamicTreeCut.

The results of enrichment in Clarivate Pathway Maps were used to visualize the frequently modulated TGF-β signaling pathways. The top enriched pathway maps specific to TGF-β signaling were used to reconstruct a network model, which describes interactions between the TGF-β signaling molecules and miRNA targets. To do this, we programmatically traced molecular signaling connections between the TGF-β signaling molecules and miRNAs involved in these pathway maps, derived linear cascades, and combined them into a network. The layout of the network was manually adjusted and visualized using Clarivate proprietary Pathway Maps Creator tool. We visualized genes known to be associated with neuronal processes, neurodegeneration, and neurotoxicity in the reconstructed TGF-β network. We derived neurodegeneration-relevant terms from the collection of disease biomarkers from Cortellis Drug Discovery Intelligence (CDDI), neurotoxicity-relevant terms from Clarivate MetaCore toxic pathologies ontology, and neurological processes from the Gene Ontology (GO) Processes. An extensive collection of CDDI neurodegeneration-relevant biomarkers was filtered to select only gene-term annotations with the highest trust. To do this, we selected only biomarkers which were annotated and “recommended/approved” or “late studies in humans” in CDDI, which corresponds to either FDA-approved biomarkers or biomarkers derived from studies with over 500 individuals. To identify GO processes which were potentially relevant for neurodegeneration, we specifically identified children of terms: “positive regulation of nervous system process,” “regulation of nervous system process,” “negative regulation of nervous system process,” “regulation of neuronal action potential,” and “regulation of neuron projection regeneration.” In addition, we selected all GO terms which contained the text “neuro.” Genes associated with these terms were mapped on the network.

##### Identification of key miRNAs via ingenuity pathway analysis

In order to identify the most relevant targets and key miRNAs of interest and to better elucidate the interactions between miRNAs, targets, and the pathways, we performed further analyses using the ingenuity pathway analysis (IPA) software (Qiagen), as outlined in Supplementary Figure 1. First, mature miRNA annotations (within the IPA database) that corresponded to our 39 significant miRNAs were manually curated. Then, over 10 000 experimentally validated mRNA targets of the 39 significant mature miRNAs were identified via miRNA target filter analysis. Of those mRNA targets, we selected only those 402 mRNAs that were associated with “neurological disease” category curated in the IPA database. The full list of terms included in this category is available in Supplementary Table 3. The filtered set of mRNA targets was then assessed in the IPA Pathway tool for (1) their roles as biomarkers for neurological diseases, or (2) their involvement in neurologically relevant canonical pathways or in the commonly significant pathways identified from mirPath analysis. The full list of biomarker and pathway terms used in this assessment can be found in Supplementary Table 3. We excluded mRNA targets that (1) did not display any relationships to the selected biomarkers or canonical pathways, or (2) were associated only with 1 specific neurological condition. For the remaining mRNA targets, we identified the miRNAs regulating those mRNA targets from the list of our 39 significant miRNAs. Then the changes in the expression of the mRNA targets were predicted based on the observed expression changes of the miRNAs.

## RESULTS

###  

#### Characterizing Morphological and Cytotoxic Effects of Classic Neurotoxicants on hiPSC-Derived Neurons

**Figure 1.**
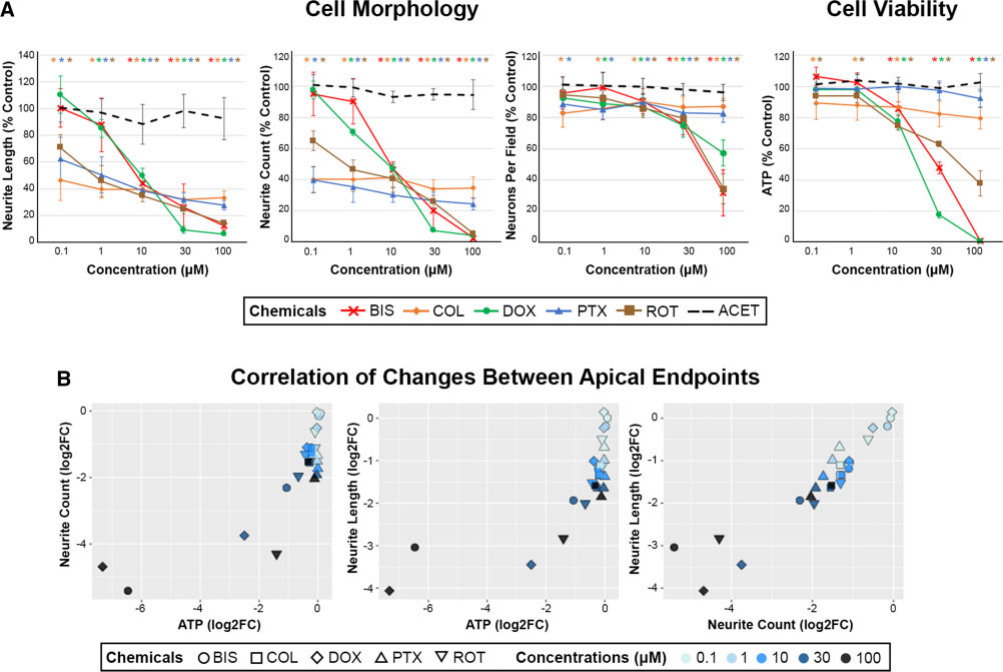
Effects of neurotoxic chemicals on cell morphology and viability in human-induced pluripotent stem cell (hiPSC)-neurons. A, hiPSC-neurons were exposed to 0.1–100 µM of 6 chemicals (color coded figure legend) for 24 h and analyzed for cell morphology and viability endpoints via automated imaging analysis (neurite length, neurite count, and neurons per field) and ATP analysis, respectively. Data are presented as mean ± SD, with color-coded asterisks * denoting significant changes compared with vehicle control (Dunnett’s test, *p* < .05). Adapted with permission from Cohen and Tanaka (2018). B, The changes in cell morphology and viability endpoints were assessed for the correlation across endpoints using Pearson’s correlation analysis. Each correlation plot represents log_2_ fold changes of the different apical endpoints on each axis, and correlation coefficient (*r* values) and significance (*p* values) were included within each data plot. Each chemical and concentration were denoted with a different shape or color, respectively.

A set of classic neurotoxicants (ACET [negative control], BIS, COL, DOX, PTX, and ROT), with known neurodegenerative effects *in vitro*, *in vivo*, and/or in patients on nervous system tissues, were evaluated in hiPSC-derived neurons for changes to cell morphology and cell viability after 24 h of exposure (Figure 1A). Mechanisms of neurotoxicity for each chemical are described in Supplementary Table 4. Images of the chemical response to neurons are shared in Supplementary Figure 2.

Across all tested neurotoxicants, a decline in morphological endpoints was observed at lower concentrations than those that induced significant cytotoxicity. Morphological endpoints were sensitive to detect neurodegeneration at concentrations ≤ 1 µM of COL, DOX, PTX, and ROT even with no or minimal effects on ATP levels or neurons per field (Figure 1A). Decreases in neurite count and neurite length were observed at ≥ 0.1 µM with COL, PTX, and ROT; ≥ 1 µM with DOX, and ≥ 10 µM with BIS. In contrast, ATP was decreased approximately 25–100% at ≥ 10 µM for BIS, DOX, and ROT; and neurons per field decreased approximately 40–60% at 100 µM. COL and PTX, both microtubule-targeting agents, caused significant reductions in neurite morphology endpoints with minimal effects on ATP or neurons per field up to 100 µM. These results suggest that alteration in neurite outgrowth may be a more sensitive marker for early detection of neurotoxicant insult than cell viability alone, as described in Cohen and Tanaka (2018). No effect on any endpoint was observed with ACET, the negative control. Furthermore, there was a significant correlation across apical endpoints, with more extensive morphological changes correlating to more extensive depletion of ATP (Figure 1B). Altogether, our results suggest that morphological changes can be sensitive predictors of cytotoxic effects induced by neurotoxicants, and that measuring cell viability alone may be insufficient to capture neurodegenerative effects of certain chemicals.

#### Secreted miRNA Analysis

Based on the evaluation of the morphological and viability results as described in the Characterizing Morphological and Cytotoxic Effects of Classic Neurotoxicants on hiPSC-Derived Neurons section, 2 concentrations (low and high) of each chemical were selected for miRNA analysis (Human 754 miRNA Panels), with the low concentration attributed to no or minimal effect on cell viability (≤ 20%) but with a significant decrease in neurite outgrowth (≥ 20%), and the high concentration with a significant decrease in both endpoints, with the exception of COL and PTX. Since COL and PTX had minimal changes in cell viability even up to the highest concentration tested, 100 µM was selected for the high concentration of miRNA analysis.

**Figure 2.**
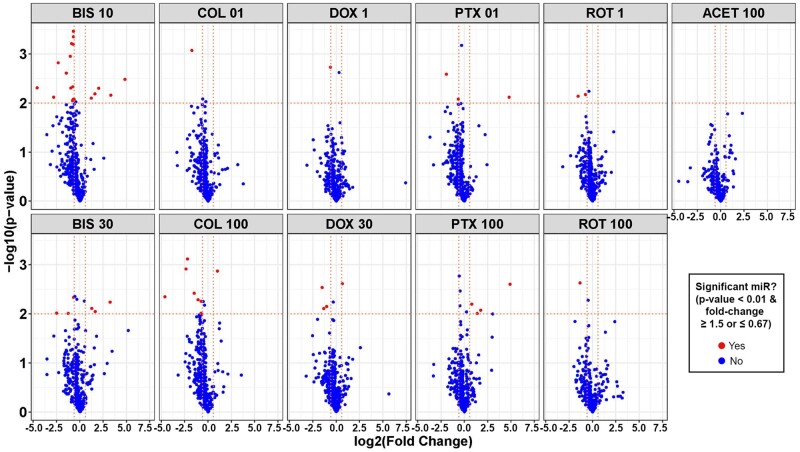
Volcano plots of secreted microRNAs (miRNAs) significantly altered in human-induced pluripotent stem cell-neurons by neurotoxic chemicals. For each chemical treatment, paired *t* tests were evaluated for each miRNA, comparing treatment versus vehicle control (DMSO) using normalized expression values, and resulting *p* values and fold change were plotted on *y*- and *x*-axis of volcano plots, respectively. Horizontal dotted lines in the plots represent a *p* value of .01 and vertical dotted lines represent a fold change of 0.67 or 1.5. Red dots represent miRNAs with fold change ≤ 0.67 or ≥ 1.5, and *p* value < .01, whereas blue dots represent miRNAs that were not significantly changed. Abbreviated chemical names are followed by concentrations (µM).

**Figure 3.**
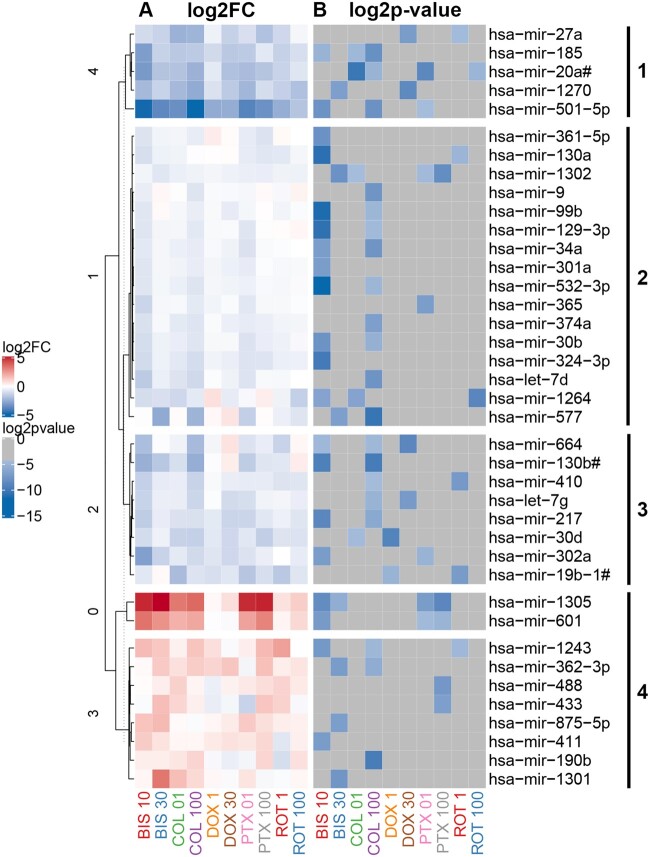
Heatmap of secreted microRNAs (miRNAs) significantly altered by chemical treatment of neurons. A total of 39 miRNAs were selected based on criteria described in methods, with fold change ≥ 1.5 or ≤ 0.67 (A, left-hand panel) and unadjusted *p* value < .01 (B, right-hand panel). miRNAs were clustered (clusters 1, 2, 3, and 4) using hierarchical clustering based on miRNA fold change values.

In Figure 2, volcano plots show miRNAs significantly altered across compound treatment groups, with significant results emphasized in red as having an unadjusted *p* value < .01 and a fold change of ≥ 1.5 or ≤ 0.67. Based on these stringent criteria, the miRNA screen identified 39 secreted miRNAs that were potentially related to decreases in neuronal cell morphology and viability parameters, which we could use to delineate the progressive stages of neurodegeneration (Figure 3). ACET did not significantly regulate any of these 39 miRNAs. The clustering (1 through 4) demonstrated in Figure 3 reflects the overall direction and intensity of change per cluster. Thus we were able to capture the general trend of alterations in miRNA expression, which may reflect the trend of the response to the toxicants. Cluster 1 mainly consisted of highly downregulated miRNAs, whereas cluster 4 included highly upregulated miRNAs. Clusters 2 and 3 were composed of mildly dysregulated miRNAs.

#### Key Signaling Pathways Underlying Chemical-Induced Neurodegeneration

The set of 39 significant miRNAs, clustered into 4 groups within Figure 3, were analyzed for their associated pathways. First, each cluster of miRNAs was assessed individually for their predicted mRNA targets and KEGG pathways significantly enriched by those targets (*p* value threshold < .05). miRNAs in clusters 1 and 4 were predicted to target genes within 8 significant pathways (Supplementary Table 5). miRNAs in clusters 2 and 3 targeted 11 and 17 significant pathways, respectively (Supplementary Table 5). Although a distinctive set of pathways was associated with each cluster (Supplementary Table 5), 3 pathways were identified to be commonly associated with all 4 clusters of significant miRNAs, as well as common across all 5 chemicals (Table 1). These pathways included: TGF-β signaling, lysine degradation, and pluripotency of stem cells. All 5 chemicals caused significant changes in 1 or more miRNAs involved in these pathways, suggesting that these pathways reflect general neurotoxicity, rather than neurotoxic response specific to an individual chemical.

**Table 1.** Pathways Commonly Enriched by Messenger RNA Targets Associated With Clusters of Significantly Altered miRNAs

Pathway	miRNAs Involved	Chemicals Involved^*a*^
TGF-β signaling	miR-27a-3p, miR-20a-3p, miR-20a-5p, miR-1305, miR-1243, miR-362-3p, miR-488-3p, miR-1301-3p, miR-361-5p, miR-130a-3p, miR-1302, miR-301a-3p, miR-374a-5p, miR-130b-3p, miR-410-3p, miR-217, miR-302a-5p, and miR-302a-3p	BIS(L, H), COL(L, H), DOX(H), PTX(L, H), and ROT(L)
Lysine degradation	miR-27a-3p, miR-20a-3p, miR-20a-5p, miR-1270, miR-488-3p, miR-875-5p, miR-1301-3p, miR-324-3p, miR-1264, miR-577, miR-130b-5p, let-7g-3p, and miR-302a-3p	BIS(L, H), COL(L, H), DOX(H), PTX(L, H), and ROT(H)
Signaling pathways regulating pluripotency of stem cells	miR-27a-3p, miR-20a-3p, miR-20a-5p, miR-1305, miR-488-3p, miR-374a-5p, let-7d-5p, miR-577, miR-664a-3p, miR-410-3p, let-7g-5p, let-7g-3p, miR-217, and miR-302a-5p	BIS(L), COL(L, H), DOX(H), PTX(L, H), and ROT(L)

Abbreviations: H, high dose; L, low dose.

a Chemicals with significant changes in 1 or more miRNAs involved.

**Figure 4.**
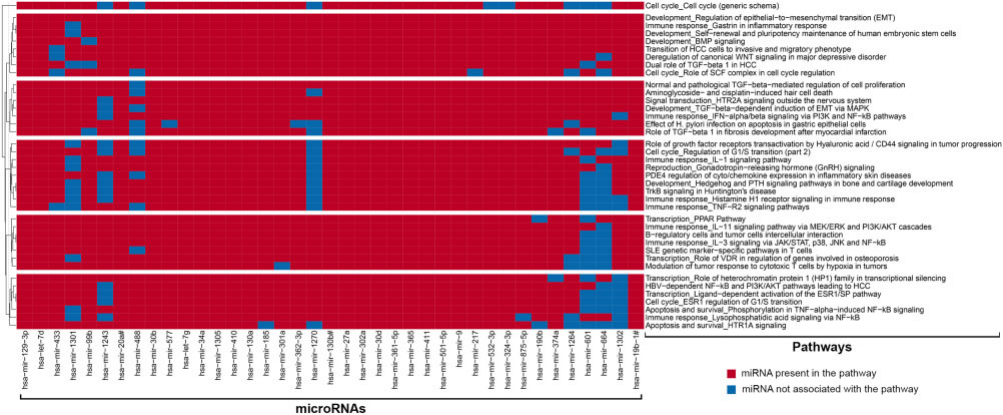
Heatmap of enriched pathway maps for significantly altered microRNAs (miRNAs). The multistep process for selection of enriched pathways, using hypergeometric distribution significance testing (significance < .001), is detailed in the Materials and Methods section. miRNAs are on the *x*-axis and red color represents presence of the targets of miRNAs in each corresponding pathway. Clustering of the pathways on the *y*-axis was performed using hierarchical clustering with average linkage and Euclidian distance.

We also performed pathway enrichment analysis using the Clarivate Pathway Maps ontology with the entire set of 39 significant miRNAs, rather than individual clusters. The Clarivate Pathway Maps ontology represents a collection of terms, each of which encompasses 3–5 signaling pathways describing particular biological mechanisms. We used 2 complementary enrichment methods, as described in Materials and Methods section, to account for potential bias from unequal number of targets per miRNA, and to avoid any overemphasis of miRNAs whose targets are poorly represented in the pathway. This allowed for a more robust selection of biological terms that were significantly enriched by the targets associated with the set of 39 significant miRNAs (Figure 4). Many of these terms were related to immune response (involving cytokines such as Tumor necrosis factor-alpha (TNF-α), interleukin (IL)-1, IL-3, and IL-11) and cell cycle regulation (regulation of G1/S transition, or role of Skp, Cullin, F-box containing (SCF) complex in cell cycle regulation). Notably, multiple terms identified within this analysis were related to TGF-β signaling pathway, which was also one of 3 commonly enriched KEGG pathways identified from the cluster-based pathway analysis. Altogether, the results across enrichment methods suggest a key role of TGF-β signaling pathway as an underlying mechanism for chemical-induced neurotoxicity.

#### Enrichment Emphasizes 5 miRNAs as Potential Biomarkers for Chemical-Induced Neurodegeneration

**Figure 5.**
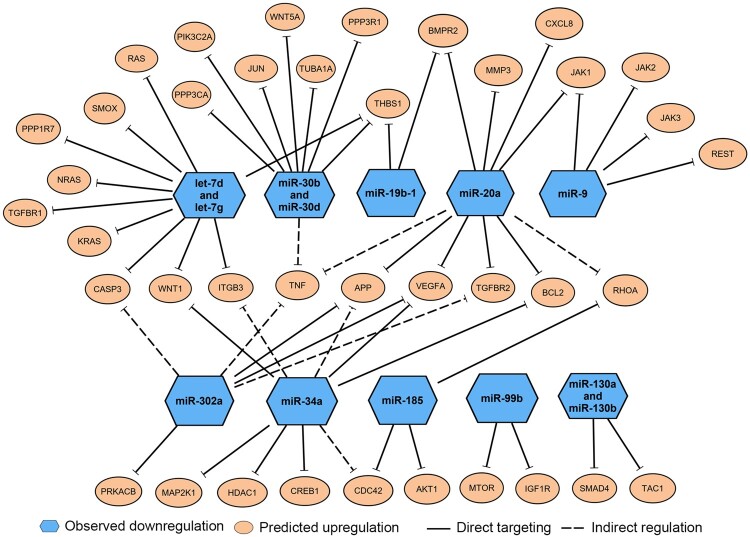
Enriched messenger RNA (mRNA) targets of 13 microRNAs (miRNAs) related to neurological diseases or neurotoxicity. Ingenuity pathway analysis microRNA Target Filter Analysis identified 38 mRNA targets (orange circles) with known association to neurological dysfunctions or pathways associated with neurotoxicity. These mRNA targets were associated with 13 downregulated miRNAs (blue hexagons), which directly target those mRNAs (solid lines) or indirectly regulate the mRNA expression (dashed lines).

Further pathway analysis using the IPA software was performed to better understand the interactions of our significant miRNAs to their mRNA targets and pathways related to neurological dysfunctions, and ultimately to identify key miRNAs and their mRNA targets that play a role in neurotoxicity. Based on the input of the 39 significant miRNAs into the IPA, a total of 402 experimentally validated and neurologically relevant mRNA targets were detected by the IPA miRNA Target Filter Analysis. Using the IPA software we further explored the roles of these mRNA targets as specific biomarkers for neurological diseases and as key molecules in canonical pathways associated with neurotoxicity. We further filtered the 402 targets down to 38 mRNA targets that were related to multiple neurological diseases and/or functions (Figure 5). Notably, many of these targets were those involved in TGF-β signaling, inflammatory response, and cell cycle, which were pathways highlighted by both enrichment analyses described earlier. Based on the final 38 mRNA targets, we mapped the relationships of the mRNA targets to their corresponding miRNAs (Figure 5). Of the original 39 significant miRNAs, 13 of the downregulated miRNAs mapped to the 38 mRNAs highlighted in Figure 5. The details of the mRNA targets and biological relationships to neurological diseases/functions associated with these 13 miRNAs were summarized in Table 2. Of the 13 miRNAs, 3 miRNAs (miR-20a, miR-30b, and miR-30d) were particularly notable because: (1) they were significantly altered by 3 neurotoxicants; (2) 1 or more targets were specifically noted as potential biomarkers for various neurological diseases; and (3) several targets were linked to the TGF-β signaling pathway, a common pathway highlighted across our enrichment processes.

**Table 2.** Enriched mRNA Targets and Biological Pathways of miRNAs Related to Neurological Diseases or Neurotoxicity

miRNA	Average Fold Change	mRNA Targets	Biomarker Association	Canonical Pathway Association	Chemicals Involved^*a*^
hsa-miR-302a	↓7.15	VEGFA, TNF, TGFBR2, APP, PRKACB, and CASP3	Multiple sclerosis, Schizophrenia, Alzheimer’s disease, Parkinson’s disease, mild cognitive impairment, multiple sclerosis, and brain cancer	Amyloid processing, neuroinflammation signaling pathway, axonal guidance signaling, synaptogenesis signaling pathway, amyotrophic lateral sclerosis signaling, neuroprotective role of THOP1 in Alzheimer’s disease, TGF-β signaling, Parkinson’s signaling, synaptic long-term potentiation, dopamine receptor signaling, neuropathic pain signaling in dorsal horn neurons, and Huntington’s disease signaling	BIS(L) and PTX(L)
hsa-miR-20a	↓3.57	MMP3, VEGFA, CXCL8, TNF, TGFBR2, APP, BMPR2, JAK1, RHOA, and BCL2	Multiple sclerosis, Schizophrenia, Alzheimer’s disease, Parkinson’s disease, mild cognitive impairment, and brain cancer	Amyloid processing, neuroinflammation signaling pathway, axonal guidance signaling, role of NANOG in mammalian embryonic stem cell pluripotency, glioblastoma multiforme signaling, glioma invasiveness signaling, synaptogenesis signaling pathway, amyotrophic lateral sclerosis signaling, neuroprotective role of THOP1 in Alzheimer’s disease, and TGF-β signaling	COL(L, H), PTX(L), and ROT(H)
hsa-let-7d and hsa-let-7g	↓2.12 and ↓2.04	WNT1, THBS1, TGFBR1, NRAS, ITGF3, CASP3, SMOX, RAS, KRAS, and PPP1R7	Brain cancer	Amyloid processing, neuroinflammation signaling pathway, axonal guidance signaling, role of NANOG in mammalian embryonic stem cell pluripotency, glioblastoma multiforme signaling, glioma invasiveness signaling, synaptogenesis signaling pathway, amyotrophic lateral sclerosis signaling, TGF-β signaling, Parkinson’s signaling, synaptic long-term potentiation, dopamine receptor signaling, dopamine degradation, and Huntington’s disease signaling	COL(H) and DOX(H)
hsa-mir-30b and hsa-miR-30d	↓1.96 and ↓1.54	TNF, WNT5A, PPP3CA, PPP3R1, JUN, TBHS1, TUBA1A, and PIK3C2A	Multiple sclerosis, Schizophrenia, Alzheimer’s disease, Parkinson’s disease, and brain cancer	Neuroinflammation signaling pathway, axonal guidance signaling, role of NANOG in mammalian embryonic stem cell pluripotency, glioblastoma multiforme signaling, amyotrophic lateral sclerosis signaling, TGF-β signaling, synaptic long-term potentiation, Huntington’s disease signaling, neuropathic pain signaling in dorsal horn neurons, glioma invasiveness signaling, synaptogenesis signaling pathway, and glioma signaling	BIS(L), COL(L, H) and DOX(L)
hsa-miR-34a	↓1.54	HDAC1, CDC42, MAP2K1, CREB1, APP, BCL2, ITGB3, VEGFA, and WNT1	Alzheimer’s disease, Parkinson’s disease, mild cognitive impairment, multiple sclerosis, and brain cancer	Amyloid processing, neuroinflammation signaling pathway, axonal guidance signaling, role of NANOG in mammalian embryonic stem cell pluripotency, glioblastoma multiforme signaling, glioma invasiveness signaling, synaptogenesis signaling pathway, amyotrophic lateral sclerosis signaling, neuroprotective role of THOP1 in Alzheimer’s disease, TGF-β signaling, synaptic long-term potentiation, neuropathic pain signaling in dorsal horn neurons, and Huntington’s disease	BIS(L) and COL(H)
hsa-miR-130a and hsa-miR-130b	↓0.355 and ↓2.358	SMAD4 and TAC1	None	Neuroprotective role of THOP1 in TGF-β signaling, neuropathic pain signaling in dorsal horn neurons and role of NANOG in mammalian embryonic stem cell pluripotency	BIS(L), COL(H), and ROT(L)
hsa-miR-185	↓1.474	RHOA, CDC42, and AKT1	None	Axonal guidance signaling, amyloid processing, neuroinflammation signaling pathway, glioma invasiveness signaling, glioblastoma multiforme signaling, role of NANOG in mammalian embryonic stem cell pluripotency, TGF-β signaling, synaptogenesis signaling pathway, glioma signaling, and Huntingtion’s disease signaling	BIS(L) and COL(L, H)
hsa-miR-9	↓0.737	JAK1, JAK2, JAK3, and REST	None	Neuroinflammation signaling pathway, role of NANOG in mammalian embryonic stem cell pluripotency, Huntington’s disease signaling, and role of Oct4 in mammalian embryonic stem cell pluripotency	COL(H)
hsa-miR-99b	↓0.690	IGF1R and MTOR	None	Synaptic long-term depression, glioblastoma multiforme signaling, synaptogenesis signaling pathway, glioma signaling, and Huntington’s disease signaling	BIS(L) and COL(H)
hsa-miR-19b-1	↓1.556	THBS1, BMPR2	Brain cancer	Neuroinflammation signaling pathway, role of NANOG in mammalian embryonic stem cell pluripotency, TGF-β signaling, and synaptogenesis signaling pathway	DOX(L) and ROT(L)

Abbreviations: H, high dose; L, low dose.

a Chemicals with significant changes in the indicated miRNA.

Focusing on these 3 downregulated miRNAs, we performed a literature review to identify prior evidence demonstrating their roles in neurological dysfunction. Prior studies demonstrated that all 3 of these miRNAs were dysregulated in various neurological conditions, suggesting that these miRNAs play key roles in neurological processes (Table 3). The expression of miR-20a was downregulated in hiPSC-derived neurons upon exposure to COL, PTX, and ROT, and was previously shown to be either up- or downregulated, depending on the model or disease type. For instance, miR-20a was upregulated in the brain of cerebral ischemia/reperfusion injury rat models (Yang *et al.*, 2021), whereas it was downregulated in the brain of equivalent mouse models (Zhong *et al.*, 2020), suggesting that directionality of miRNA response may be variable depending on the species. In clinical studies, miR-20a was upregulated in serum samples of multiple system atrophy and AD patients (Cheng *et al.*, 2015; Kume *et al.*, 2018), whereas it was downregulated in blood samples of MS patients (Cox *et al.*, 2010), implying diverse miRNA regulation across different types of neurodegenerative diseases. Notably, miR-20a inhibition in rat dorsal root ganglia (DRG) was shown to impair neurite outgrowth, emphasizing the important role miR-20a may play in neurodegeneration (Zhao *et al.*, 2021).

**Table 3.** Summary of Neurological Associations to Notable miRNAs

miRNA	Average Fold Change	Chemicals Involved	Targets	Neurological Associations	Other Relevant Findings
miR-20a	↓3.57	COL, PTX, and ROT	TGFBR2, TNF-α, RhoA, APP, BCL2, MAP3K12, MEF2D, RGMa, Neurod1, NOR-1, BMPR2, RUNX1, CXCL8, BCL2L11, CCND1, JAK1, H2AX, E2F1, and BNIP2	miR-20a upregulated in glioblastoma cells (Wei *et al.*, 2015), in brain tissues of cerebral I/R injury rat models (Yang *et al.*, 2021), in spinal cord tissues of spinal cord injury mice (Liu *et al.*, 2015), in hippocampus of epileptic rats (Feng *et al.*, 2020), in serum samples of multiple system atrophy patients (Kume *et al.*, 2018), in serum exosomes of AD patients (Cheng *et al.*, 2015), in hippocampal tissues of AD mouse model (Zhang *et al.*, 2014), and in primary culture hippocampal neurons treated with H_2_O_2_ (Zhang *et al.*, 2014) miR-20a downregulated in blood samples of treatment-naive MS patients (Cox *et al.*, 2010) and in brain tissues of mouse I/R brain injury model (Zhong *et al.*, 2020) miR-20a upregulated during the axon regeneration of DRG neurons, supporting the neurite outgrowth (Zhao *et al.*, 2021) miR-20a inhibition impairs neurite outgrowth, possibly via targeting NOR-1 miR-20a downregulated in SH-SY5Y cells and mouse NS cells during the differentiation (Aranha *et al.*, 2010; Beveridge *et al.*, 2009)	miR-20a-5p targets TGFBR-2. miR-20a-5p downregulation leads to TGFBR2-mediated TGF-β pathway activation, leading to inflammation in liver fibrosis (Fu *et al.*, 2020; Zhou *et al.*, 2015)
miR-30b	↓1.96	BIS and COL	CNR1, PAI-1, SCNA, EphB2, SIRT1, GluA2, BCL6. TNF-α, and IL6	miR-30b upregulated in hippocampal tissues of AD patients and AD rat models (Li *et al.*, 2020a; Song *et al.*, 2019), in plasma and PBMCs of PD patients (Brennan *et al.*, 2019), and in glioma cell lines (Jian *et al.*, 2019) miR-30b downregulated in serum/plasma from ALS, MS, and AD patients (Brennan *et al.*, 2019; Dong *et al.*, 2021; Liguori *et al.*, 2018), in brain samples from Schizophrenia patients (Perkins *et al.*, 2007), in serum samples of neonates with hypoxic-ischemic encephalopathy (Wang and Jia, 2021), in hypoxia-induced human brain microvascular endothelial cells (Wang and Jia, 2021), and in neuroblastoma cells treated with MPP+ (Shen *et al.*, 2020) Inhibiting miR-30b was shown to decrease the neurite length, likely mediated by miR-30b targeting Sema3A/RhoA pathway, which is an important regulator for axonal growth (Wang *et al.*, 2020)	TGF-β signaling pathway was shown to regulate the expression of miR-30s (TGF-β treatment downregulated miR-30s, and this is potentially mediated via Smad2)—shown in human umbilical vein endothelial cells (Ciavarella *et al.*, 2021) and human podocytes (Shi *et al.*, 2013)
miR-30d	↓1.54	COL and DOX	USP22, BECN1, ATG5, TNF-α, PARP, BCL6, GNAI2 and PLECKHA7	miR-30d upregulated in blood samples from bipolar disease patients (Maffioletti *et al.*, 2016) and in plasma samples from Huntington’s disease patients (Diez-Planelles *et al.*, 2016) miR-30d downregulated in CSF samples from AD patients (Brennan *et al.*, 2019), in methyl mercury-exposed immortalized human neural progenitor cells (Wang *et al.*, 2016), in brain tissue of a hypoxia ischemia rat model and cerebral I/R mouse model (Jin *et al.*, 2021; Zhao *et al.*, 2017) and in peripheral blood samples from acute ischemic stroke patients (Jiang *et al.*, 2018)	
miR-1305	↑4.826	BIS and PTX	TGFB2, POLR3G, Smad4, RUNX2 (Chen and Liu, 2017), Wnt2, TXNRD1 (Li *et al.*, 2020b), and MDM2 (Cai *et al.*, 2019)	No prior evidence of association with neurological diseases/processes	miR-1305, which targets POLR3G (downstream target of OCT4 and NANOG, pluripotency markers), regulates the differentiation of human embryonic stem cells (Overexpression of miR-1305 was shown to induce cell differentiation, whereas miR-1305 knockdown supports the maintenance of pluripotency of human embryonic stem cells; Jin *et al.*, 2016) miR-1305 mimic significantly reduced the expression and activity of TGF-β2 in bladder cancer cells (Su *et al.*, 2020) miR-1305 was shown to target Smad4 in TGF-β pathway, and Smad4 was shown to be critical for chondrogenesis (Zhang *et al.*, 2017)
miR-1243	↑1.604	BIS, COL, and ROT	SMAD2, SMAD4, and Angiomotin (Liu *et al.*, 2020)	No prior evidence of association with neurological diseases/processes	Shown to increase the sensitivity to gemcitabine in pancreatic cancer by inhibiting epithelial-mesenchymal transition, possibly mediated through its targeting of SMAD2/4 which is involved in TGF-β signaling (Hiramoto *et al.*, 2017)

**Figure 6.**
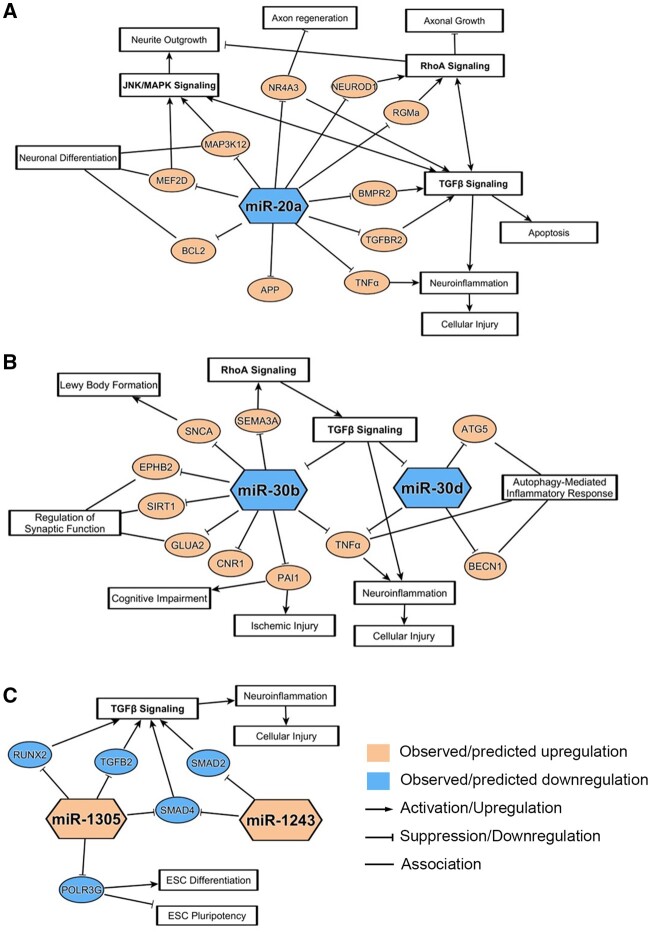
Neurodegenerative pathways associated with notable down- and upregulated microRNAs (miRNAs). miRNA-messenger RNA (mRNA) associations and related pathways were derived from ingenuity pathway analysis and/or literature review for the 3 notable downregulated miRNAs (A, B) and the 2 notable upregulated miRNAs (C). mRNA pathways depicted here have a correlation to pathways involved in neuronal dysregulations and chemical-induced neurotoxicity.

Similarly, miR-30b and -30d were previously shown to be dysregulated in several neurological diseases, similar to what we observed in hiPSC-derived neurons treated with neurotoxicants. Particularly, miR-30b, which was downregulated upon exposure to BIS and COL in our study, was also downregulated in a wide variety of neurological conditions, from neurodegenerative diseases to brain tumors to psychiatric diseases. In patients, miR-30b expression was downregulated in serum or plasma samples from amyotrophic lateral sclerosis (ALS), MS, and AD patients, and brain samples from Schizophrenia patients (Brennan *et al.*, 2019; Dong *et al.*, 2021; Liguori *et al.*, 2018; Perkins *et al.*, 2007). Such findings suggest that miR-30b may be a universal marker for neuronal dysregulation and neurotoxicity. Interestingly, miR-30b was found to be significantly upregulated in hippocampal tissues of AD patients and rat models (Li *et al.*, 2020a; Song *et al.*, 2019), and in plasma samples of Parkinson’s disease patients (Brennan *et al.*, 2019), further supporting the fact that miRNA regulation can be differentially regulated based on the model, disease context, or even sample matrix. In vitro, similar to our study, inhibition of miR-30b resulted in a decrease in neurite length of rat primary sensory neurons (Wang *et al.*, 2020). miR-30d, which was downregulated upon exposure to COL and DOX in our study, was also downregulated in blood samples of acute ischemic stroke patients and in CSF samples of AD patients (Brennan *et al.*, 2019; Jiang *et al.*, 2018). *In vitro*, exposure of methyl mercury to immortalized human neural progenitor cells resulted in downregulation of miR-30d upon neurotoxicant exposure, similar to what we observed in our study (Wang *et al.*, 2016). The proposed pathways of neurodegeneration associated with these 3 key downregulated miRNAs, miR-20a, -30b, and -30d, are shown in Figures 6A and 6B.

Next, we conducted a literature search focused on the associations between significantly upregulated miRNAs and neurologically relevant pathways or processes. Of the 10 miRNAs upregulated within cluster 4 of Figure 3, only miR-1305, miR-601, and miR-1243 were significantly altered by at least 2 chemicals and 3 or more exposure groups. We found that these 3 miRNAs were much less extensively reported on by prior research, and that there were no studies demonstrating the direct association between these miRNAs and neurological dysfunctions. Although these miRNAs were not highlighted within the IPA analysis, the literature suggested 2 miRNAs, miR-1305 and miR-1243, as potential regulators of TGF-β signaling, implying that these 2 upregulated miRNAs may be involved in neurotoxic processes through the TGF-β pathway (Figure 6C).

**Figure 7.**
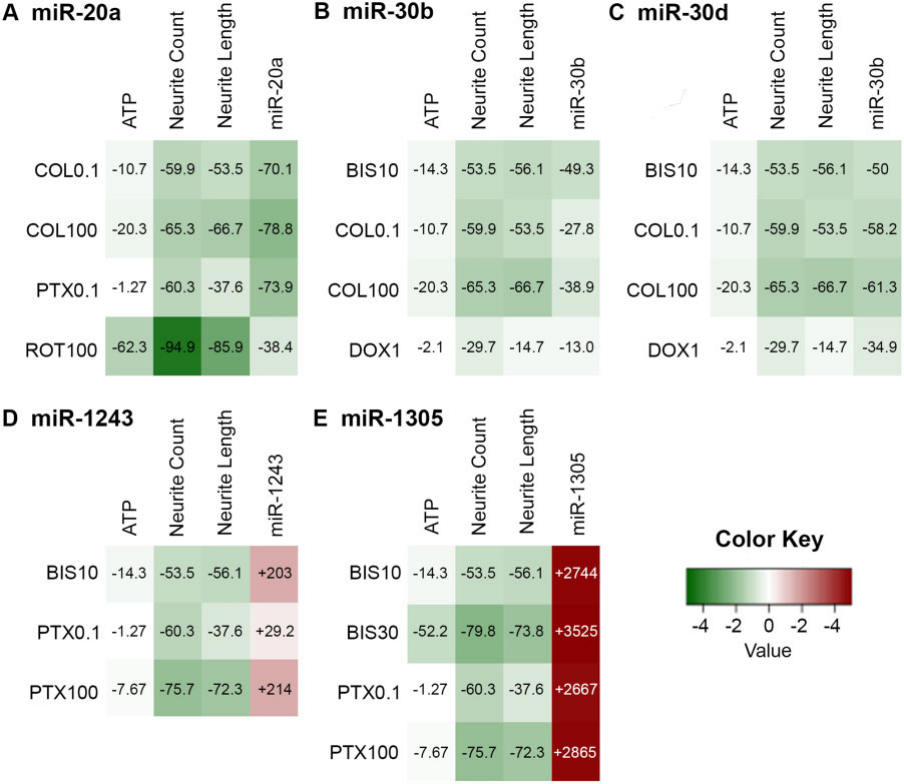
Association between changes in microRNA (miRNA) expression and apical endpoints for notable miRNAs. The changes in the 3 apical endpoints (adenosine triphosphate, neurite count, and neurite length) were compared with the changes in the 5 notable miRNAs (3 downregulated [A–C] and 2 upregulated miRNAs [D, E]). The values overlayed in the heatmaps correspond to percent changes in each of the apical endpoints and miRNAs after treatment with chemicals. Abbreviated chemical names are followed by concentrations (µM).

For these 5 most notable miRNAs (miR-20a, miR-30b, miR-30d, miR-1305, and miR-1243), we compared the chemical-induced changes in their miRNA expression to the changes in cell viability and morphology endpoints, in order to assess how well the miRNA expression reflected the apical observations (Figure 7). The percentage changes in the expression of the 5 miRNAs generally followed similar trends as the percentage changes in apical endpoints, particularly the morphological changes in neurites (decreases in neurite count and/or neurite length). For the most part, chemicals that induced more notable changes in the neurite count or neurite length also caused more extensive changes in the miRNA expression. Overall, these results suggest that alterations in levels of the 5 notable miRNAs were reflective of morphological abnormalities in hiPSC-derived neurons, which further supports the role of these miRNAs as potential biomarkers for neurodegeneration.

#### Molecular Network of TGF-β Signaling in Relation to 5 Key miRNAs

**Figure 8.**
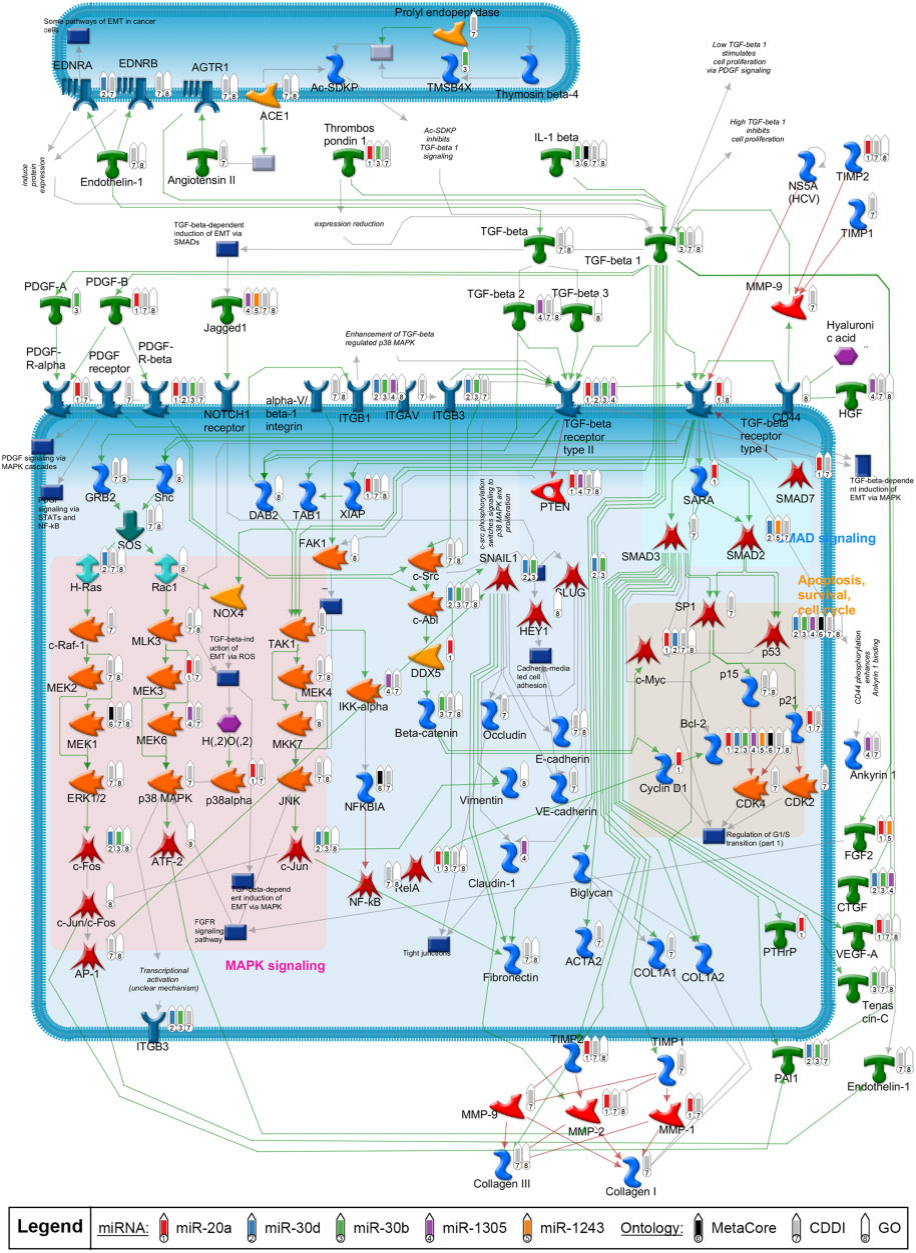
Causal network of transforming growth factor-beta (TGF-β) signaling pathways enriched with microRNA (miRNA) targets. TGF-β signaling cascades were programmatically derived from corresponding top enriched Clarivate pathway maps as described in methods. Green and red arrows represent activating or inhibiting connection between proteins, respectively. Gray arrows represent a connection with an unclear effect or technical connections (eg, interactions between an object which represents multiple proteins or individual proteins). Thermometers in the network mark the targets of the top 5 notable miRNAs (thermometers 1 through 5), as well as the targets with neurological associations identified from the different ontologies (thermometers 6 through 8).

Using the pathway enrichment results from Clarivate Pathway Maps, we constructed a network model to display the relationships between TGF-β signaling, the 5 most notable miRNAs (miR-20a, miR-30b, miR-30d, miR-1305, and miR-1243), and the neurological pathways (Figure 8). Numerous molecules within the TGF-β network were shown to be targeted by at least one of the 5 miRNAs. Two molecules that were targeted by the highest number of miRNAs were TGF-β receptor type II and the apoptosis regulator BCL-2, suggesting these 2 targets are highly correlative to neurodegenerative processes. Additionally, many of the genes in the network were involved in neurological or neurotoxic processes (within either Clarivate MetaCore Toxic Pathologies Ontology or the GO) or identified as biomarkers for neurodegenerative conditions (within CDDI Database; Figure 8 and  Supplementary Table 6). These results further affirm the role of the TGF-β pathway as the underlying mechanisms of neurotoxicity upon chemical exposure.

## DISCUSSION

Pharmaceuticals, drugs of abuse, industrial chemicals, and environmental toxicants have all been associated with direct or indirect detrimental damage to nervous tissue. Early detection and diagnosis of chemical-induced neurodegenerative adverse events are therefore critical for human safety. To date, there are no validated predictive biomarkers for drug-induced neurodegeneration in patients. This is partially due to limited translatability of animal models used for safety biomarker discovery to humans (DeFelipe *et al.*, 2002; Roberts *et al.*, 2015). Here, we assessed neurotoxic chemicals of different modes of toxicities in hiPSC-derived neurons to identify secreted miRNA biomarkers that correlated with chemical-induced neurodegeneration. We demonstrated (1) dose-dependent changes in cell viability and morphology endpoints in hiPSC-derived neurons following neurotoxicant exposure, and a strong correlation across these apical endpoints; (2) the combination of these apical endpoints could sensitively delineate the stages of neurotoxic insult and select appropriate concentrations for miRNA screening; (3) 39 miRNAs, identified by stringent criteria for significance, were modulated by ≥ 1 chemical treatment; (4) 5 secreted miRNAs (miR-20a, -30b, -30d, -1243, and -1305) were notable based on their relationship to key neurological mRNA targets and pathways, and their correlation to morphological changes induced by multiple neurotoxicants (*n* ≥ 2); and (5) TGF-β signaling was a common pathway highlighted across our enrichment analyses, which suggests a relationship of TGF-β signaling to neurological dysfunction.

Neurotoxicants of different chemical classes and mechanisms of action were selected to elucidate secreted miRNA biomarkers and signaling pathways commonly reflective of neurodegeneration (chemical mechanisms and neurological associations highlighted in Supplementary Table 4). As expected from the chemicals’ diverse mechanisms of action, each chemical modulated different numbers and sets of the 39 miRNAs, but there were also miRNAs commonly identified across multiple chemicals (Figure 3). Interestingly, many of the miRNAs did not display dose-dependent changes, but rather, low and high concentrations of each chemical tended to show distinct patterns of miRNA regulation, alluding to a shift in the pattern of miRNA regulation at progressive stages of neurodegeneration. In some cases, low and high concentrations altered the same miRNA in the opposite direction. Similar observations were made in a study with human neuroblastoma cells, which showed that different sets of genes and pathways were modulated by noncytotoxic versus cytotoxic concentrations of manganese (Fernandes *et al.*, 2019). Likewise, our data suggest that low concentrations of the neurotoxicants studied may affect miRNAs which regulate genes and pathways specific to neurite morphology or outgrowth, whereas the high concentrations may trigger modulations in miRNAs secondary to overt cytotoxicity.

The combination of the IPA analyses and the literature review of neurological associations for the panel of significant miRNAs, enabled us to identify 5 most notable miRNAs as potential biomarkers for chemical-induced neurodegeneration: 3 downregulated miRNAs (miR-20a, miR-30b, and miR-30d) and 2 upregulated miRNAs (miR-1305 and miR-1243). The degree of changes in these 5 miRNAs correlated well to the extent of morphological decline in the hiPSC-derived neurons following chemical exposure, which suggests that these miRNAs can serve as indirect measures for assessing the extent of chemical-induced neurotoxicity. Additionally, for all 5 of these miRNAs at least 1 chemical modulated the same miRNA at both low and high concentrations, suggesting these miRNAs are relevant independent of stage of neurodegeneration.

The 3 downregulated miRNAs, miR-20a, -30b, and -30d, have extensive associations to neurological diseases. These miRNAs have been shown to be modulated in blood samples of patients across different types of neurodegenerative diseases (Brennan *et al.*, 2019; Cheng *et al.*, 2015; Cox *et al.*, 2010; Diez-Planelles *et al.*, 2016; Dong *et al.*, 2021; Jiang *et al.*, 2018; Kume *et al.*, 2018; Li *et al.*, 2020a; Liguori *et al.*, 2018; Maffioletti *et al.*, 2016; Perkins *et al.*, 2007). In our study, these miRNAs were secreted into the media following neuronal dysregulation by at least 2 chemicals. We specifically focused on secreted biomarkers in vitro, since secreted factors would be most likely to translate to detection of miRNAs in fluids (serum, CSF, and urine) of animals or humans. Additionally, these miRNAs were previously shown to be altered in a variety of nonclinical systems, such as human neuroblastoma and neural progenitor cells, and nonclinical species including mice and rats under neurotoxic conditions (Aranha *et al.*, 2010; Beveridge *et al.*, 2009; Feng *et al.*, 2020; Jeyaseelan *et al.*, 2008; Jian *et al.*, 2019; Jin *et al.*, 2021; Liu *et al.*, 2015; Shen *et al.*, 2020; Song *et al.*, 2019; Wang and Jia, 2021; Wang *et al.*, 2016, 2020; Wei *et al.*, 2015; Yang *et al.*, 2021; Zhang *et al.*, 2014; Zhao *et al.*, 2017, 2021; Zhong *et al.*, 2020). This suggests a strong translation of effect across species and increases our confidence that these notable miRNAs may be predictive biomarkers of chemical-induced neurodegeneration in nonclinical safety studies and in humans.

Two upregulated miRNAs, miR-1305, and -1243, have been less extensively investigated, with no prior evidence elucidating their roles in neurological processes or dysfunctions, but the expression of these miRNAs have been confirmed in human brain (Hiramoto *et al.*, 2017; Ludwig *et al.*, 2016; Qian *et al.*, 2021; Su *et al.*, 2020; Zhang *et al.*, 2017). These miRNAs were of particular interest, because they were highly upregulated across multiple chemicals, and shown to target mRNA molecules related to TGF-β signaling (TGF-β2, SMAD2, or SMAD4).

In addition to the 5 miRNA biomarker candidates that were deemed notable based on our analyses, other miRNAs may also warrant further investigation, given that more miRNAs might have been identified as notable with a broadener set of chemicals. Therefore, we expanded our literature review within the 39 significant miRNAs to include miRNAs that were modulated by more than 1 chemical, detailed in Supplementary Table 7. Additionally, miR-9 and let-7, which were among the 13 miRNAs highlighted by the IPA analysis, may also warrant further investigation. miR-9, which was significantly downregulated upon exposure to COL in our study, is highly expressed in both developing and adult brains and was shown to play key roles in neurogenesis and neuronal differentiation (Radhakrishnan and Alwin Prem Anand, 2016). Also, miR-9 was identified as a potential biomarker for neurotoxicity in recent studies by Imam *et al.* (2018) and Das Gupta *et al.* (2021). The levels of miR-9a-3p and miR-9a-5p were shown to be significantly elevated in CSF of rats exposed to the neurotoxicant trimethyltin (Imam *et al.*, 2018). In a separate study, miR-9 expression was significantly elevated in plasma of both traumatic brain injury patients and rodent models, correlating with the disease severity (Das Gupta *et al.*, 2021). Both studies support the role of miR-9 as a predictor of neuronal injury. Similarly, let-7 family was identified as a key salivary biomarker that could signify traumatic brain injury in male athletes (Di Pietro *et al.*, 2021). In our study, 2 of let-7 family members, let-7d and let-7g, were significantly dysregulated by exposure to COL and DOX. The circulating levels of these 2 miRNAs were also shown to be significantly altered in patients with AD, ALS, or attention-deficit/hyperactivity disorder, suggesting their roles as signatures of various types of neurological conditions (Aharon *et al.*, 2020; Liguori *et al.*, 2018; Wu *et al.*, 2015).

The collective enrichment analyses identified multiple pathways of interest that are regulated by our key miRNAs and may drive chemical-induced neurodegeneration (Figure 4). Of those pathways, TGF-β signaling had the strongest association to neurodegenerative processes (Figure 8). TGF**-**β signaling regulates multiple cellular processes, including cell growth, apoptosis, differentiation, morphogenesis, and tissue homeostasis (Dennler *et al.*, 2002; Massague, 2012). Importantly, TGF-β canonical (SMAD-dependent) and noncanonical signaling pathways were shown to be involved in distinct neural or neurodevelopmental processes, such as neurogenesis, neuronal proliferation, differentiation, survival, and microglial activation (Massague, 2012). A loss of TGF**-**β1 resulted in a widespread degeneration of neurons and microgliosis in neonatal mice (Brionne *et al.*, 2003).

Additionally, *in vitro*, *in vivo*, and clinical data have demonstrated critical roles for TGF-β signaling in various neurological injuries or disorders, including chemical-induced hippocampal injury, traumatic brain injury, Parkinson’s, AD, and ALS (Bruccoleri *et al.*, 1998; Buss *et al.*, 2008; Munoz *et al.*, 2020; Seth *et al.*, 2017). For example, inhibiting TGF-β signaling prevented a pesticide carbofuran-mediated neurotoxicity in both rat hippocampal neural stem cell cultures and rat hippocampal tissues (Seth *et al.*, 2017). Also, the imbalance of TGF-β signaling was suggested as a key factor in the etiology and progression of ALS (Galbiati *et al.*, 2020; Peters *et al.*, 2017). Altered serum levels of TGF-β1 and proinflammatory cytokines were noted in ALS patients (Peters *et al.*, 2017). The reduction in TGF-β signaling at an early stage of ALS is thought to block the neuroprotective effects of TGF-β, whereas at later stages of the disease, the over-activation of the TGF-β signaling promotes microglial activation and hence proinflammatory responses which cause further neurotoxicity (Galbiati *et al.*, 2020). Altogether, TGF-β signaling has a wide spectrum of roles in regulating the homeostasis of the nervous system (Hiew *et al.*, 2021).

Since an integral aspect of our analysis was to compare miRNA results across studies with diverse test systems, it’s important to highlight the complexities of comparing miRNA findings across studies with different patient disease backgrounds or differences between *in vivo* and *in vitro* disease models. The type of test system (human, *in vivo*, or *in vitro*), species, sex, genetic background, chemical exposure, disease conditions, and biological matrices could all contribute to diversity in miRNA responses when comparing across studies (Corrales *et al.*, 2021; Harrill *et al.*, 2016; Razak *et al.*, 2013; Wang *et al.*, 2009). We believe that an integrative approach, utilizing both chemically induced and disease model miRNA data is necessary to identify relevant miRNA biomarkers that will be useful for predictive safety in patients. Additionally, it is important to acknowledge the limitation of using a monoculture of hiPSC-derived neuronal cells, which is lacking the interplay between neurons, glial, and other cells that are important in promoting neuronal cell health and function and in regulating miRNA signaling. Yet, using a relatively pure population of neurons to evaluate chemical-induced toxicity may have less inherent variability than animal models or patients, as tissues other than the nervous system may be impacted and consequently affect the regulation of secreted miRNAs.

In summary, we have identified a novel panel of 39 secreted miRNA biomarkers for detecting neurotoxic insults in hiPSC-neurons. These miRNAs, along with their highly correlated targets and signaling pathways, may be important tools in developing our understanding of chemical-induced neurodegeneration and reducing neurotoxicity-induced drug attrition. Future studies will be necessary to assess the *in vitro* to *in vivo* translation of our miRNA biomarker candidates, which will provide insights into the utility of our miRNAs as fluidic biomarkers to detect neurotoxicity of drug candidates in nonclinical safety studies. 

## SUPPLEMENTARY DATA

Supplementary data are available at *Toxicological Sciences* online.

## DECLARATION OF CONFLICTING INTERESTS

D.Y., J.D.C., L.L., and L.S. acknowledge that they are employed by and own stock in Takeda Pharmaceutical Company Limited.

## Supplementary Material

kfac011_Supplementary_Data
