# Supplementary material for: Profiling Secreted miRNA Biomarkers of Chemical-Induced Neurodegeneration in Human iPSC-Derived Neurons
Source: Toxicol Sci. 2022 Feb 4;186(2):221–41. doi: 10.1093/toxsci/kfac011 (PMC8963304; doi:10.1093/toxsci/kfac011)

**Profiling Secreted miRNA Biomarkers of Chemical-Induced Neurodegeneration in Human iPSCDerived Neurons**

Dahea You^a,1^, Jennifer D. Cohen^a,1,*^, Olga Pustovalova^b^, Lauren Lewis^c^, Lei Shen^d^

^a^Drug Safety Research & Evaluation, Takeda Development Center Americas, Inc., San Diego, CA USA

^b^Clarivate, Philadelphia, PA, USA

^c^Drug Safety Research & Evaluation, Takeda Development Center Americas, Inc., Cambridge, MA USA

^d^Data Science Institute, Takeda Development Center Americas, Inc., Cambridge, MA USA

^1^D. You and J.D. Cohen contributed equally to this work as first authors

**Supplemental Tables**

**Supplemental Table 1. Summary of miRNAs Significantly Dysregulated Upon Neurotoxicant Exposure** *(*Included as a separate file)*

**Supplemental Table 2. Annotations of significantly altered miRNA used for mirPath analyses**

| **Cluster 1** | **Cluster 2** | **Cluster 3** | **Cluster 4** |
| --- | --- | --- | --- |
| hsa-miR-27a-3p  hsa-miR-27a-5p  hsa-miR-185-3p  hsa-miR-185-5p  hsa-miR-20a-3p  hsa-miR-20a-5p  hsa-mir-1270  hsa-mir-501-5p | hsa-miR-361-5p  hsa-miR-130a-5p  hsa-miR-130a-3p  hsa-miR-1302  hsa-miR-99b-5p  hsa-miR-99b-3p  hsa-miR-129-1-3p  hsa-miR-129-2-3p  hsa-miR-34a-3p  hsa-miR-34a-5p  hsa-miR-301a-3p  hsa-miR-301a-5p  hsa-miR-532-3p  hsa-miR-374a-3p  hsa-miR-374a-5p  hsa-miR-30b-3p  hsa-miR-30b-5p  hsa-miR-324-3p  hsa-let-7d-3p  hsa-let-7d-5p  hsa-miR-1264  hsa-miR-577 | hsa-miR-664a-3p  hsa-miR-664a-5p  hsa-miR-130b-5p  hsa-miR-130b-3p  hsa-miR-410-3p  hsa-miR-410-5p  hsa-let-7g-5p  hsa-let-7g-3p  hsa-miR-217  hsa-miR-30d-5p  hsa-miR-30d-3p  hsa-miR-302a-5p  hsa-miR-302a-3p  hsa-miR-19b-3p  hsa-miR-19b-1-5p | hsa-miR-1305  hsa-miR-601  hsa-miR-1243  hsa-miR-362-3p  hsa-miR-488-3p  hsa-miR-488-5p  hsa-miR-433-5p  hsa-miR-433-3p  hsa-miR-875-5p  hsa-miR-411-3p  hsa-miR-411-5p  hsa-miR-190b  hsa-miR-1301-5p  hsa-miR-1301-3p |

**Supplemental Table 3. List of neurological disease, biomarker and canonical pathway terms included in the IPA analyses**

| **Neurological Disease Terms** | Disease of central nervous system; disease of peripheral nervous system; disease of sensory nervous system; neurological disorder of absolute anatomical region; neurological disorder; encephalopathy; degenerative central nervous system disorder; congenital neurological disorder; metabolic disorder of nervous system; autosomal recessive neurological disorder; pediatric-onset neurological disease; cardiovascular disorder of nervous system; white matter abnormality; sporadic neurological disorder, hematological disorder of nervous system; thalamic disease; demyelination; inflammatory disorder of nervous system; gangliosidosis; dermatological disorder of nervous system; respiratory disorder of nervous system; Waardenburg-Shah syndrome neurological variant; infantile-onset multisystem neurologic, endocrine and pancreatic disease; endocrine system of nervous system; Perrault syndrome with neurological disease; neurological Wilson disease; global neurological disorder; psychiatric Wilson disease; neonatal onset multi-system inflammatory disease with mild or severe mental retardation |
| --- | --- |
| **Biomarker Terms** | Alzheimer’s disease, Brain cancer, Mild cognitive impairment, multiple sclerosis, Parkinson’s disease, Schizophrenia |
| **Canonical Pathway Terms** | Amyloid processing, Amyotrophic lateral sclerosis signaling, Axonal guidance signaling, Dopamine degradation, Dopamine receptor signaling, Glioblastoma Multiforme signaling, Glioma invasiveness signaling, Glioma signaling, Huntington’s disease signaling, Neuroinflammation signaling pathway, Neuropathic pain signaling in dorsal horn neurons, Neuroprotective role of THOP1 in Alzheimer’s disease, Parkinson’s signaling, Role of NANOG in mammalian embryonic stem cell pluripotency, Role of Oct4 in mammalian embryonic stem cell pluripotency, Synaptic long-term depression, Synaptic long-term potentiation, Synaptogenesis signaling pathway, TGF-beta signaling, |

**Supplemental Table 4. Mechanisms of Neurotoxicity by Tested Chemicals**

| **Chemical** | **Classification** | **Mechanism(s) of Action** | **Neurological association(s)** |
| --- | --- | --- | --- |
| Bisindolylmaleimide-1 | Investigative chemical | Protein kinase C inhibition (Toullec et al. 1991) | Inhibition of neurite outgrowth (Das et al. 2004; Nakanishi et al. 2006; Robinette et al. 2011) |
| Colchicine | Therapeutic for gout | Binds to tubulin resulting in inhibition of microtubule assembly therefore preventing mitosis and disrupting axoplasmic transport (Mundy and Tilson 1990) | Microtubule depolymerization (Vandecandelaere et al. 1997) |
| Doxorubicin | Chemotherapeutic | Induces ROS production, DNA damage, and mitochondrial membrane depolarization in neurons (Du et al. 2021; Ongnok et al. 2020) | Cognitive impairment and CNS toxicity (El-Agamy et al. 2019; Shaker et al. 2021) |
| Paclitaxel | Chemotherapeutic | Interference with microtubule depolymerization and repolymerization by binding to the length of microtubules which stabilizing them (Gornstein and Schwarz 2014) | Microtubule hyper-stabilization (Gornstein and Schwarz 2017); axonal degeneration (Yang et al. 2009) |
| Rotenone | Environmental toxicant | Mitochondrial complex 1 (MC1) inhibition (Chance et al. 1963) and microtubule depolymerization (Brinkley et al. 1974) | Microtubule depolymerization (Ren and Feng 2007) |

**Supplemental Table 5. Pathways Enriched by Targets Associated with Each Cluster of Significantly Altered miRNAs.** For each individual miRNA cluster (Cluster 1 thru 4) of 39 significant miRNAs highlighted in Figure 3, mirPath analysis shows KEGG pathways involved with multiple miRNAs and chemicals.

| **Cluster** | **KEGG Pathway^1^** | **p-value^2^** | **# genes^3^** | **# miRNAs^4^** | **miRNAs^4^** | **Chemicals^5^** |
| --- | --- | --- | --- | --- | --- | --- |
| **1** | Proteoglycans in cancer | 1.97E-05 | 57 | 4 | miR-27a-3p, miR-185-3p, miR-20a-3p, miR-20a-5p | COL(L,H), DOX(H), PTX(L) |
|  | Axon guidance | 3.94E-05 | 34 | 3 | miR-185-3p, miR-185-5p, miR-20a-5p | COL(L,H), PTX(L) |
|  | TGF-beta signaling pathway | 0.00020 | 28 | 3 | miR-27a-3p, miR-20a-3p, miR-20a-5p | COL(L), DOX(H), PTX(L) |
|  | Lysine degradation | 0.00092 | 16 | 4 | miR-27a-3p, miR-20a-3p, miR-20a-5p, miR-1270 | BIS(H), COL(L), DOX(H), PTX(L) |
|  | Signaling pathways regulating pluripotency of stem cells | 0.0020 | 37 | 3 | miR-27a-3p, miR-20a-3p, miR-20a-5p | COL(L), DOX(H), PTX(L) |
|  | Circadian entrainment | 0.018 | 26 | 2 | miR-27a-3p, miR-185-3p | COL(H), DOX(H) |
|  | Thyroid hormone signaling pathway | 0.025 | 18 | 2 | miR-185-5p, miR-20a-3p | COL(L,H), PTX(L) |
|  | ErbB signaling pathway | 0.029 | 29 | 2 | miR-27a-3p, miR-20a-5p | COL(L), DOX(H), PTX(L) |
| **2** | Prion diseases | 0 | 1 | 2 | miR-130a-3p, miR-301a-3p | BIS(L) |
|  | Glycosphingolipid biosynthesis - lacto and neolacto series | 5.33E-15 | 10 | 6 | miR-361-5p, miR-130a-5p, miR-129-2-3p, miR-34a-3p, miR-34a-5p, miR-374a-3p | BIS(L), COL(H) |
|  | Biosynthesis of unsaturated fatty acids | 1.46E-06 | 5 | 2 | miR-1302, miR-374a-5p | BIS(H), COL(H) |
|  | TGF-beta signaling pathway | 2.30E-06 | 29 | 5 | miR-361-5p, miR-130a-3p, miR-1302, miR-301a-3p, miR-374a-5p | BIS(L,H), COL(H) |
|  | Mucin type O-Glycan biosynthesis | 0.00012 | 9 | 3 | miR-532-3p, miR-30b-5p, let-7d-5p | BIS(L), COL(H) |
|  | Signaling pathways regulating pluripotency of stem cells | 0.00096 | 46 | 3 | miR-374a-5p, let-7d-5p, miR-577 | BIS(H), COL(H) |
|  | ECM-receptor interaction | 0.00099 | 8 | 2 | let-7d-5p, miR-1264 | COL(H), ROT(H) |
|  | Glycosaminoglycan biosynthesis - heparan sulfate / heparin | 0.0016 | 9 | 4 | miR-361-6p, miR-130a-5p, miR-99b-5p, miR301a-5p | BIS(L,H), COL(H) |
|  | Lysine degradation | 0.021 | 10 | 3 | miR-324-3p, miR-1264, miR-577 | BIS(L,H), COL(H), ROT(H) |
|  | FoxO signaling pathway | 0.022 | 48 | 5 | miR-130a-3p, miR-301a-3p, miR-374a-5p, miR-30b-5p, miR-577 | BIS(L,H), COL(H) |
|  | Proteoglycans in cancer | 0.030 | 44 | 3 | miR-361-5p, miR-130a-5p, miR-1264 | BIS(L), ROT(H) |
| **3** | Prion diseases | 0 | 2 | 3 | miR-130b-3p, miR-410-3p, miR-30d-3p | BIS(L), COL(H), DOX(L), ROT(L) |
|  | Signaling pathways regulating pluripotency of stem cells | 2.06E-10 | 57 | 6 | miR-664a-3p, miR-410-3p, let-7g-5p, let-7g-3p, miR-217, miR-302a-5p | BIS(L), DOX(H), ROT(L) |
|  | TGF-beta signaling pathway | 3.24E-10 | 33 | 5 | miR-130b-3p, miR-410-3p, miR-217, miR-302a-5p, miR-302a-3p | BIS(L), COL(L), ROT(L) |
|  | Proteoglycans in cancer | 1.59E-08 | 75 | 7 | miR-664a-3p, miR-664-5p, miR-410-3p, let-7g-3p, miR-217, miR-302a-3p, miR-19b-3p | BIS(L), DOX(H), ROT(L) |
|  | ECM-receptor interaction | 2.32E-07 | 8 | 1 | let-7g-5p | DOX(H) |
|  | FoxO signaling pathway | 3.28E-05 | 56 | 6 | miR-664a-3p, miR-130b-3p, miR-410-3p, let-7g-3p, miR-217, miR-30d-5p | BIS(L), COL(H), DOX(L,H), ROT(L) |
|  | Metabolism of xenobiotics by cytochrome P450 | 6.34E-05 | 6 | 2 | miR-130b-3p, miR-410-5p | BIS(L), COL(H), ROT(L) |
|  | Estrogen signaling pathway | 0.00028 | 20 | 3 | miR-302a-5p, miR-302a-3p, miR-19b-3p | BIS(L), ROT(L) |
|  | Mucin type O-Glycan biosynthesis | 0.0024 | 9 | 3 | let-7g-5p, miR-30d-5p, miR-302a-5p | BIS(L), DOX(L,H) |
|  | Lysine degradation | 0.0026 | 11 | 3 | miR-130b-5p, let-7g-3p, miR-302a-3p | BIS(L), COL(H), DOX(H) |
|  | Glioma | 0.0093 | 19 | 4 | miR-130b-5p, miR-130b-3p, miR-217, miR-302a-5p | BIS(L), COL(H) |
|  | Axon guidance | 0.017 | 42 | 3 | let-7g-3p, miR-30d-5p, miR-302a-3p | BIS(L), DOX(L,H) |
|  | mTOR signaling pathway | 0.019 | 25 | 4 | miR-664a-3p, miR-130b-3p, miR-410-3p, miR-19b-3p | BIS(L), COL(H), DOX(H), ROT(L) |
|  | Wnt signaling pathway | 0.023 | 21 | 2 | miR-664a-5p, let-7g-3p | DOX(H) |
|  | Morphine addiction | 0.023 | 26 | 4 | miR-664a-3p, let-7g-3p, miR-30d-5p, miR-302a-5p | BIS(L), DOX(L,H) |
|  | GABAergic synapse | 0.029 | 17 | 3 | miR-664a-3p, let-7g-3p, miR-19b-1-5p | DOX(H), ROT(L) |
|  | Gap junction | 0.035 | 25 | 4 | miR-664a-3p, miR-130b-3p, miR-30d-5p, miR-19b-3p | BIS(L), COL(H), DOX(L,H), ROT(L) |
| **4** | Hippo signaling pathway | 2.57E-13 | 43 | 4 | miR-1305, miR-488-3p, miR-433-5p, miR-411-3p | BIS(L), PTX(L,H) |
|  | TGF-beta signaling pathway | 1.11E-07 | 27 | 5 | miR-1305, miR-1243, miR-362-3p, miR-488-3p, miR-1301-3p | BIS(L,H), PTX(L,H) |
|  | Glycosphingolipid biosynthesis - lacto and neolacto series | 6.37E-07 | 4 | 3 | miR-488-3p, miR-190b, miR-1301-5p | BIS(H), COL(H), PTX(H) |
|  | Morphine addiction | 3.49E-05 | 6 | 2 | miR-1243, miR-411-5p | BIS(L) |
|  | Mucin type O-Glycan biosynthesis | 6.39E-05 | 9 | 2 | miR-1305, miR-875-5p | BIS(L,H), PTX(L,H) |
|  | Signaling pathways regulating pluripotency of stem cells | 0.0078 | 41 | 2 | miR-1305, miR-488-3p | BIS(L), PTX(L,H) |
|  | Lysine degradation | 0.025 | 8 | 3 | miR-488-3p, miR-875-5p, miR-1301-3p | BIS(H), PTX(H) |
|  | Long-term depression | 0.039 | 21 | 2 | miR-1305, miR-488-3p | BIS(L), PTX(L,H) |

^1^Pathways enriched by the predicted targets of each cluster of miRNAs

^2^Probability that the examined pathway is significantly enriched with gene targets of at least one miRNA in each cluster

^3^Number of genes that are involved in each pathway and are predicted targets of miRNAs

^4^miRNAs (from each cluster) that are associated with indicated pathway

^5^Chemicals with significant changes in one or more miRNAs involved in each pathway; H: High dose, L: Low dose

**Supplemental Table 6. Neurologically Relevant Associations of TGF-β Network Molecules** *(*Included as a separate file)*

**Supplemental Table 7. Summary of Neurological Associations to miRNAs Significantly Dysregulated in hiPSC-Derived Neurons by Two or More Chemicals**

| **miRNA** | **Avg. Fold Change** | **Chemicals Involved** | **Neurological Associations and Other Relevant Findings** |
| --- | --- | --- | --- |
| let-7g | ↓2.04 | COL, DOX | - let-7g-3p up-regulated in CSF exosomes of PD patients (Gui et al. 2015) - let-7g-3p down-regulated in plasma of the rhesus monkey model of ischemic stroke (Chen et al. 2021) - let-7g-5p up-regulated in serum samples of AD patients (Poursaei et al. 2021) and in plasma of the rhesus monkey model of ischemic stroke (Chen et al. 2021) - let-7g-5p down-regulated in extracellular vesicles of AD patients (Aharon et al. 2020) and in blood samples of glioblastoma patients (Dong et al. 2014) - let-7g down-regulated in PBMCs of MS patients (Martinelli-Boneschi et al. 2012), in serum samples of Schizophrenia patients (Shi et al. 2012), in human primary brain microvascular endothelial cells under oxygen glucose deprivation (Bernstein et al. 2020), and in mouse models of AD (Chum et al. 2021) and ischemia/reperfusion stroke (Bernstein et al. 2020) - TGFBR1 and SMAD2 are the targets of let-7g (Liao et al. 2014; Zhou et al. 2015) |
| miR-19b-1 | ↓2.94 | DOX, ROT | - miR-19b up-regulated in peripheral blood of Schizophrenia patients (Horai et al. 2020), in tumor tissues of glioma patients (Jia et al. 2013), in human glioma cell lines (Jia et al. 2013), and in serum and brain tissues of a rat model of post-traumatic stress (Balakathiresan et al. 2014) - miR-19b down-regulated in serum of Parkinson’s disease patients (Botta-Orfila et al. 2014; Cao et al. 2017b) - A single nucleotide polymorphism in miR-19b may confer susceptibility to autism spectrum disorder (Toma et al. 2015) - miR-19b targets TGFBR2 (Borzi et al. 2019; Lakner et al. 2012) |
| miR-27a | ↓2.50 | DOX, ROT | - miR-27a up-regulated in monocytes of ALS patients (Ravnik-Glavac and Glavac 2020), in hippocampal tissues of epileptic rats (Lu et al. 2019), and in primary rodent hippocampal neurons exposed to kainic acid or sevoflurane (Lu et al. 2019; Lv et al. 2017) - miR-27a down-regulated in PBMCs of PD patients (Fazeli et al. 2020), in serum exosomes of ALS patients (Ravnik-Glavac and Glavac 2020), in CSF of AD patients (Sala Frigerio et al. 2013), in brain tissues of rodent TBI models (Sabirzhanov et al. 2014; Sun et al. 2017), in spinal cord of rat cerebral ischemia/reperfusion model (Li et al. 2015), and in in vitro model of cerebral ischemia/reperfusion model (Li et al. 2021) - miR-27a targets TGFBR1, SMAD2 and SMAD4 (Chae et al. 2017; Fang et al. 2018b) - miR-27a increases the protein expression of TGF-β and SMAD7 (Bai et al. 2019) - TGF-β treatment increases the expression of miR-27a-5p in human NK cells (Regis et al. 2017) |
| miR-34a | ↓1.54 | BIS, COL | - miR-34a up-regulated in serum, plasma, PBMCs, or extracellular fluid of major depressive disorder, Schizophrenia, Alzheimer’s disease, Parkinson’s disease, aneurysmal subarachnoid hemorrhage, Multiple Sclerosis, ischemic stroke patients (Liang and Lou 2016; Piotrzkowska et al. 2021; Supriya et al. 2020; van den Berg et al. 2020), in brain tissues of alcohol use disorder patients (Santos-Bezerra et al. 2021), in blood and brain tissues of mouse cerebral ischemia injury model (Liang and Lou 2016), in apoptotic hippocampal tissues of mice treated with ketamine (Jiang et al. 2014), and in rat pheochromocytoma cell lines exposed to MPP+ (Rostamian Delavar et al. 2018) - miR-34a down-regulated in serum, plasma, or small extracellular vesicles of Alzheimer’s disease and Parkinson’s disease patients (Grossi et al. 2021; Li et al. 2020b; van den Berg et al. 2020), in tumor tissues of neuroblastoma and mesial temporal lobe epilepsy patients (Organista-Juarez et al. 2019; Wang et al. 2021c), in rat spinal cord injury model (Jian et al. 2020), in mouse microglia treated with LPS (Jian et al. 2020), in primary mouse cortical neurons and mouse neural crest-derived cells exposed to amyloid beta (Li et al. 2020b), and in primary rat dorsal root ganglion after sciatic nerve injury (Zou et al. 2020) - Overexpression of miR-34a inhibited the proliferation, migration and invasion of human neuroblastoma cells (Li and Chen 2019) - Inhibition of miR-34a increased the number of branches of mouse primary cortical neurons whereas overexpression of miR-34a led to a reduction of total dendritic length and branch numbers (Agostini et al. 2011) - miR-34a targets TGFBR2, SMAD4 and TGIF2 (Feili et al. 2018; Ma et al. 2015; Pan et al. 2021; Qiao et al. 2015) |
| miR-99b | ↓1.61 | BIS, COL | - miR-99b-5p up-regulated in plasma samples of down syndrome patients (Salvi et al. 2019) and in spinal cord of mouse spinal cord injury model (Cao et al. 2017a) - miR-99b down-regulated in plasma samples of acute cerebral infarct patients (Wu et al. 2020), in tumor tissues of glioma patients (Zhang et al. 2016a), and in brain tissues of rat status epilepticus model (Sun et al. 2013) |
| miR-129-3p | ↓1.64 | BIS, COL | - miR-129-3p down-regulated in human glioblastoma tumor tissues and glioblastoma cell lines (Fang et al. 2018a), and in rat primary neuronal cells exposed to glucose fluctuation (Wang et al. 2021a) - Overexpression of miR-129-3p led to overmigration of neurons in mouse embryonic brains (Wu et al. 2019) |
| miR-130a | ↓1.85 | BIS, ROT | - miR-130a up-regulated in serum of autism spectrum disorder and attention deficit-hyperactivity disorder patients (Mundalil Vasu et al. 2014; Zadehbagheri et al. 2019), in brain tissues of rat cerebral ischemia injury models (Deng et al. 2020; Wang et al. 2020; Wang et al. 2018), in lumbar tissues of rat spinal cord injury model (Yao et al. 2021), in primary rat BMECs under oxygen glucose deprivation (Wang et al. 2018), and in BV2 microglia exposed to LPS (Yao et al. 2021) - miR-130a down-regulated in serum or plasma of narcolepsy and acute inflammatory stroke patients (Holm et al. 2014; Jin and Xing 2018), in human neuroblastoma cells treated with amyloid-beta 1-42 (Wang et al. 2021d), in hippocampus of mouse Alzheimer’s disease model (Wang et al. 2021d), and in rat primary astrocytes and pericytes under oxygen glucose deprivation (Wang et al. 2018) - miR-130a was shown to reduce the neurite length of rat primary cortical neurons (Zhang et al. 2016c) - miR-130a targets TGFBR1 and TGFBR2 (Liu et al. 2021; Wang et al. 2017b) |
| miR-130b | ↓5.13 | BIS, COL | - miR-130b up-regulated in blood of Schizophrenia patients (Wei et al. 2015), in tumor tissues of glioma patients (Gu et al. 2018; Li et al. 2017), in human glioma cell lines (Gu et al. 2018), and in mouse primary hippocampal neurons treated with H_2_O_2_ (Zhang et al. 2014) - miR-130b down-regulated in in vivo mouse cerebral ischemic/reperfusion injury model and in human neuroblastoma cells (Liu et al. 2020) and primary rat astrocytes under oxygen glucose deprivation (Zheng et al. 2017) (in vitro models of cerebral ischemia/reperfusion injury model) - Overexpression of miR-130b increased the proliferation of mouse primary embryonic neural progenitor cells (Gong et al. 2013) |
| miR-185 | ↓2.78 | BIS, COL | - miR-185 up-regulated in PBMCs of ADHD patients (Sanchez-Mora et al. 2019) - miR-185 down-regulated in plasma of glioma patients (Tang et al. 2015), in brain tissues of the rat HIE model (Xiong et al. 2020) and in brain tissues of rats receiving rotenone treatment (Rahimmi et al. 2019) - miR-185 is involved in the 22q11.2 microdeletion which is the strongest known genetic factor for Schizophrenia (Forstner et al. 2014) - miR-185 targets TGFBR1 and TGFBR2 (Zhu et al. 2020a) |
| miR-217 | ↓2.78 | BIS, COL | - miR-217 up-regulated in tumor tissues of glioblastoma patients (Wang et al. 2017a), in human glioblastoma cell lines (Wang et al. 2017a), in human neuroblastoma cell lines treated with MPP+ (Wang et al. 2019b), in the rat model of cerebral ischemia/reperfusion injury (Shi et al. 2020b), and in rat primary cortical neurons under oxygen glucose deprivation (Rao et al. 2019; Shi et al. 2020b) - miR-217 down-regulated in tumor tissues of glioma and medulloblastoma patients (Kumar et al. 2018; Zhu et al. 2016), in human glioma cell lines (Zhu et al. 2016), and in human neuroblastoma cell lines under oxygen glucose deprivation (Yi et al. 2020) |
| miR-302a | ↓7.15 | BIS, PTX | - miR-302a down-regulated in blood samples of ischemic stroke patients (Zhu et al. 2020b), in tumor tissues of glioma patients (Ma et al. 2017; Zhong et al. 2021), and in human glioma cell lines (Ma et al. 2017; Zhong et al. 2021) - miR-302 was reported to be critical for embryonic viability and proper neural tube closure and neuronal differentiation (Parchem et al. 2015) |
| miR-362-3p | ↑2.41 | BIS, COL | - miR-362-3p down-regulated in tumor tissues of glioblastoma and astrocytoma patients (Kheirollahi et al. 2017; Shi et al. 2020a), in human glioblastoma cell line (Shi et al. 2020a), in rodent cerebral ischemia/ reperfusion models (Liu et al. 2010; Wang et al. 2021b), in the in vitro rat model of cerebral ischemia/reperfusion injury (Wang et al. 2021b), and in spinal cord tissues of the rat spinal cord injury model (Hu et al. 2019) - miR-362-3p targets SMAD which is involved in TGF-β signaling (Cheng et al. 2021) |
| miR-410 | ↓1.69 | COL, ROT | - miR-410 up-regulated in rat neural stem cells treated with sevoflurane (Zhang et al. 2020) - miR-410 down-regulated in tumor samples of glioma patients (Wang et al. 2019a) and high-risk neuroblastoma patients (Gattolliat et al. 2011), in serum samples of newborns with hypoxic-ischemic encephalopathy (Meng et al. 2021), in brain tissues of the rat model of hypoxic-ischemia (Xiao et al. 2020), and in pheochromocytoma cell lines (Xiao et al. 2020) - Overexpression of miR-410 inhibits the neuronal differentiation of mouse embryonic stem cells (Tsan et al. 2016) - TGFBR2 is a target of miR-410-3p (Li et al. 2020a) |
| miR-501-5p | ↓25.00 | BIS, COL, PTX | *NOTE that there were no relevant findings published regarding miR-501-5p whereas numerous studies about miR-501-3p were found. Hence the findings about miR-501-3p were summarized in this table.*   - miR-501-3p up-regulated in serum samples of AD patients (Hara et al. 2017) - miR-501-3p down-regulated in brain tissues of AD patients (Hara et al. 2017; Li and Cai 2021; Takousis et al. 2019) and in white matter tissues of a mouse model of the cerebral hypoperfusion-induced vascular cognitive impairment (Toyama et al. 2018) - miR-501-3p targets TGFBR3 (Yin et al. 2019) |
| miR-532-3p | ↓1.61 | BIS, COL | - miR-532-3p down-regulated in CSF samples of AD patients (van Harten et al. 2015) and in brain tissues and plasma of the rat cerebral ischemic/reperfusion model (Mao et al. 2020) |
| miR-577 | ↓5.00 | BIS, COL | - miR-577 down-regulated in tumor tissues of glioma patients and in human glioma cell lines (Fan et al. 2021; Wei et al. 2018; Zhang et al. 2016b) - TGF-β treatment increases the expression of miR-577 in human gastric cancer cells (Luo et al. 2019) |
| miR-601 | ↑9.94 | BIS, PTX | - miR-601 up-regulated in tumor tissues of pediatric brain cancer (Braoudaki et al. 2014) |
| miR-664 | ↑1.57 | BIS, COL, DOX | - miR-664 up-regulated in brain tissues of AD and autism spectrum disorder patients (Ander et al. 2015; Li and Cai 2021) - miR-664 down-regulated in brain tissues of AD patients (Henriques et al. 2020) and in PBMCs of Schizophrenia patients (Yu et al. 2015) - miR-664a-5p was shown to promote neuronal differentiation (Watanabe et al. 2018) |
| miR-1264 | ↓2.56 | BIS, COL, ROT | - miR-1264 up-regulated in serum samples of a mouse model of ischemic stroke and intracerebral hemorrhage (Uhlmann et al. 2017), and in human neuroblastoma cells infected with Zika virus (Bagasra et al. 2021) |
| miR-1270 | ↓2.60 | BIS, DOX | - miR-1270 down-regulated in human glioblastoma cell lines and in tumor tissues of glioblastoma multiforme patients (Wei et al. 2019) |
| miR-1302 | ↓1.59 | BIS, COL, PTX | - No relevant findings have been published. |

**References:**

Agostini M, Tucci P, Steinert JR, Shalom-Feuerstein R, Rouleau M, Aberdam D, Forsythe ID, Young KW, Ventura A, Concepcion CP et al. 2011. Microrna-34a regulates neurite outgrowth, spinal morphology, and function. Proc Natl Acad Sci U S A. 108(52):21099-21104.

Aharon A, Spector P, Ahmad RS, Horrany N, Sabbach A, Brenner B, Aharon-Peretz J. 2020. Extracellular vesicles of alzheimer's disease patients as a biomarker for disease progression. Mol Neurobiol. 57(10):4156-4169.

Ander BP, Barger N, Stamova B, Sharp FR, Schumann CM. 2015. Atypical mirna expression in temporal cortex associated with dysregulation of immune, cell cycle, and other pathways in autism spectrum disorders. Mol Autism. 6:37.

Bagasra O, Shamabadi NS, Pandey P, Desoky A, McLean E. 2021. Differential expression of mirnas in a human developing neuronal cell line chronically infected with zika virus. Libyan J Med. 16(1):1909902.

Bai Y, Liu Y, Jin S, Su K, Zhang H, Ma S. 2019. Expression of microrna27a in a rat model of osteonecrosis of the femoral head and its association with tgfbeta/smad7 signalling in osteoblasts. Int J Mol Med. 43(2):850-860.

Balakathiresan NS, Chandran R, Bhomia M, Jia M, Li H, Maheshwari RK. 2014. Serum and amygdala microrna signatures of posttraumatic stress: Fear correlation and biomarker potential. J Psychiatr Res. 57:65-73.

Bernstein DL, Gajghate S, Reichenbach NL, Winfield M, Persidsky Y, Heldt NA, Rom S. 2020. Let-7g counteracts endothelial dysfunction and ameliorating neurological functions in mouse ischemia/reperfusion stroke model. Brain Behav Immun. 87:543-555.

Borzi C, Calzolari L, Ferretti AM, Caleca L, Pastorino U, Sozzi G, Fortunato O. 2019. C-myc shuttled by tumour-derived extracellular vesicles promotes lung bronchial cell proliferation through mir-19b and mir-92a. Cell Death Dis. 10(10):759.

Botta-Orfila T, Morato X, Compta Y, Lozano JJ, Falgas N, Valldeoriola F, Pont-Sunyer C, Vilas D, Mengual L, Fernandez M et al. 2014. Identification of blood serum micro-rnas associated with idiopathic and lrrk2 parkinson's disease. J Neurosci Res. 92(8):1071-1077.

Braoudaki M, Lambrou GI, Giannikou K, Milionis V, Stefanaki K, Birks DK, Prodromou N, Kolialexi A, Kattamis A, Spiliopoulou CA et al. 2014. Microrna expression signatures predict patient progression and disease outcome in pediatric embryonal central nervous system neoplasms. J Hematol Oncol. 7:96.

Brinkley BR, Barham SS, Barranco SC, Fuller GM. 1974. Rotenone inhibition of spindle microtubule assembly in mammalian cells. Exp Cell Res. 85(1):41-46.

Cao F, Liu T, Sun S, Feng S. 2017a. The role of the mir-99b-5p/mtor signaling pathway in neuroregeneration in mice following spinal cord injury. Mol Med Rep. 16(6):9355-9360.

Cao XY, Lu JM, Zhao ZQ, Li MC, Lu T, An XS, Xue LJ. 2017b. Microrna biomarkers of parkinson's disease in serum exosome-like microvesicles. Neurosci Lett. 644:94-99.

Chae DK, Ban E, Yoo YS, Kim EE, Baik JH, Song EJ. 2017. Mir-27a regulates the tgf-beta signaling pathway by targeting smad2 and smad4 in lung cancer. Mol Carcinog. 56(8):1992-1998.

Chance B, Williams GR, Hollunger G. 1963. Inhibition of electron and energy transfer in mitochondria. I. Effects of amytal, thiopental, rotenone, progesterone, and methylene glycol. J Biol Chem. 238:418-431.

Chen J, Zhao H, Huang Y, Li Y, Fan J, Wang R, Han Z, Yang Z, Wu L, Wu D et al. 2021. Dysregulation of principal circulating mirnas in non-human primates following ischemic stroke. Front Neurosci. 15:738576.

Cheng HP, Huang CJ, Tsai ML, Ong HT, Cheong SK, Choo KB, Chiou SH. 2021. Microrna-362 negatively and positively regulates smad4 expression in tgf-beta/smad signaling to suppress cell migration and invasion. Int J Med Sci. 18(8):1798-1809.

Chum PP, Hakim MA, Behringer EJ. 2021. Cerebrovascular microrna expression profile during early development of alzheimer's disease in a mouse model. J Alzheimers Dis.

Das KP, Freudenrich TM, Mundy WR. 2004. Assessment of pc12 cell differentiation and neurite growth: A comparison of morphological and neurochemical measures. Neurotoxicol Teratol. 26(3):397-406.

Deng W, Fan C, Zhao Y, Mao Y, Li J, Zhang Y, Teng J. 2020. Microrna-130a regulates neurological deficit and angiogenesis in rats with ischaemic stroke by targeting xiap. J Cell Mol Med. 24(18):10987-11000.

Dong L, Li Y, Han C, Wang X, She L, Zhang H. 2014. Mirna microarray reveals specific expression in the peripheral blood of glioblastoma patients. Int J Oncol. 45(2):746-756.

Du J, Zhang A, Li J, Liu X, Wu S, Wang B, Wang Y, Jia H. 2021. Doxorubicin-induced cognitive impairment: The mechanistic insights. Front Oncol. 11:673340.

El-Agamy SE, Abdel-Aziz AK, Esmat A, Azab SS. 2019. Chemotherapy and cognition: Comprehensive review on doxorubicin-induced chemobrain. Cancer Chemother Pharmacol. 84(1):1-14.

Fan X, Liu M, Fei L, Huang Z, Yan Y. 2021. Circfoxm1 promotes the proliferation, migration, invasion, and glutaminolysis of glioblastoma by regulating the mir-577/e2f5 axis. Bosn J Basic Med Sci.

Fang DZ, Wang YP, Liu J, Hui XB, Wang XD, Chen X, Liu D. 2018a. Microrna-129-3p suppresses tumor growth by targeting e2f5 in glioblastoma. Eur Rev Med Pharmacol Sci. 22(4):1044-1050.

Fang F, Huang B, Sun S, Xiao M, Guo J, Yi X, Cai J, Wang Z. 2018b. Mir-27a inhibits cervical adenocarcinoma progression by downregulating the tgf-betari signaling pathway. Cell Death Dis. 9(3):395.

Fazeli S, Motovali-Bashi M, Peymani M, Hashemi MS, Etemadifar M, Nasr-Esfahani MH, Ghaedi K. 2020. A compound downregulation of srrm2 and mir-27a-3p with upregulation of mir-27b-3p in pbmcs of parkinson's patients is associated with the early stage onset of disease. PLoS One. 15(11):e0240855.

Feili X, Wu S, Ye W, Tu J, Lou L. 2018. Microrna-34a-5p inhibits liver fibrosis by regulating tgf-beta1/smad3 pathway in hepatic stellate cells. Cell Biol Int. 42(10):1370-1376.

Forstner AJ, Basmanav FB, Mattheisen M, Bohmer AC, Hollegaard MV, Janson E, Strengman E, Priebe L, Degenhardt F, Hoffmann P et al. 2014. Investigation of the involvement of mir185 and its target genes in the development of schizophrenia. J Psychiatry Neurosci. 39(6):386-396.

Gattolliat CH, Thomas L, Ciafre SA, Meurice G, Le Teuff G, Job B, Richon C, Combaret V, Dessen P, Valteau-Couanet D et al. 2011. Expression of mir-487b and mir-410 encoded by 14q32.31 locus is a prognostic marker in neuroblastoma. Br J Cancer. 105(9):1352-1361.

Gong X, Zhang K, Wang Y, Wang J, Cui Y, Li S, Luo Y. 2013. Microrna-130b targets fmr1 and regulates embryonic neural progenitor cell proliferation and differentiation. Biochem Biophys Res Commun. 439(4):493-500.

Gornstein E, Schwarz TL. 2014. The paradox of paclitaxel neurotoxicity: Mechanisms and unanswered questions. Neuropharmacology. 76 Pt A:175-183.

Gornstein EL, Schwarz TL. 2017. Neurotoxic mechanisms of paclitaxel are local to the distal axon and independent of transport defects. Exp Neurol. 288:153-166.

Grossi I, Radeghieri A, Paolini L, Porrini V, Pilotto A, Padovani A, Marengoni A, Barbon A, Bellucci A, Pizzi M et al. 2021. Microrna34a5p expression in the plasma and in its extracellular vesicle fractions in subjects with parkinson's disease: An exploratory study. Int J Mol Med. 47(2):533-546.

Gu JJ, Fan KC, Zhang JH, Chen HJ, Wang SS. 2018. Suppression of microrna-130b inhibits glioma cell proliferation and invasion, and induces apoptosis by pten/akt signaling. Int J Mol Med. 41(1):284-292.

Gui Y, Liu H, Zhang L, Lv W, Hu X. 2015. Altered microrna profiles in cerebrospinal fluid exosome in parkinson disease and alzheimer disease. Oncotarget. 6(35):37043-37053.

Hara N, Kikuchi M, Miyashita A, Hatsuta H, Saito Y, Kasuga K, Murayama S, Ikeuchi T, Kuwano R. 2017. Serum microrna mir-501-3p as a potential biomarker related to the progression of alzheimer's disease. Acta Neuropathol Commun. 5(1):10.

Henriques AD, Machado-Silva W, Leite REP, Suemoto CK, Leite KRM, Srougi M, Pereira AC, Jacob-Filho W, Nobrega OT, Brazilian Aging Brain Study G. 2020. Genome-wide profiling and predicted significance of post-mortem brain microrna in alzheimer's disease. Mech Ageing Dev. 191:111352.

Holm A, Bang-Berthelsen CH, Knudsen S, Kornum BR, Modvig S, Jennum P, Gammeltoft S. 2014. Mirna profiles in plasma from patients with sleep disorders reveal dysregulation of mirnas in narcolepsy and other central hypersomnias. Sleep. 37(9):1525-1533.

Horai T, Boku S, Okazaki S, Otsuka I, Ratta-Apha W, Mouri K, Yamaki N, Hirata T, Hishimoto A. 2020. Mir-19b is elevated in peripheral blood of schizophrenic patients and attenuates proliferation of hippocampal neural progenitor cells. J Psychiatr Res. 131:102-107.

Hu Y, Liu Q, Zhang M, Yan Y, Yu H, Ge L. 2019. Microrna-362-3p attenuates motor deficit following spinal cord injury via targeting paired box gene 2. J Integr Neurosci. 18(1):57-64.

Jia Z, Wang K, Zhang A, Wang G, Kang C, Han L, Pu P. 2013. Mir-19a and mir-19b overexpression in gliomas. Pathol Oncol Res. 19(4):847-853.

Jian YP, Dong SJ, Xu SS, Fan J, Liu WJ, Shao XW, Li T, Zhao SH, Wang YG. 2020. Microrna-34a suppresses neuronal apoptosis and alleviates microglia inflammation by negatively targeting the notch pathway in spinal cord injury. Eur Rev Med Pharmacol Sci. 24(3):1420-1427.

Jiang XL, Du BX, Chen J, Liu L, Shao WB, Song J. 2014. Microrna-34a negatively regulates anesthesia-induced hippocampal apoptosis and memory impairment through fgfr1. Int J Clin Exp Pathol. 7(10):6760-6767.

Jin F, Xing J. 2018. Circulating mir-126 and mir-130a levels correlate with lower disease risk, disease severity, and reduced inflammatory cytokine levels in acute ischemic stroke patients. Neurol Sci. 39(10):1757-1765.

Kheirollahi M, Moodi M, Ashouri S, Nikpour P, Kazemi M. 2017. Evaluation of mir-362 expression in astrocytoma of human brain tumors. Adv Biomed Res. 6:129.

Kumar V, Kumar V, Chaudhary AK, Coulter DW, McGuire T, Mahato RI. 2018. Impact of mirna-mrna profiling and their correlation on medulloblastoma tumorigenesis. Mol Ther Nucleic Acids. 12:490-503.

Lakner AM, Steuerwald NM, Walling TL, Ghosh S, Li T, McKillop IH, Russo MW, Bonkovsky HL, Schrum LW. 2012. Inhibitory effects of microrna 19b in hepatic stellate cell-mediated fibrogenesis. Hepatology. 56(1):300-310.

Li F, Li F, Chen W. 2020a. Propofol inhibits cell proliferation, migration, and invasion via mir-410-3p/transforming growth factor-beta receptor type 2 (tgfbr2) axis in glioma. Med Sci Monit. 26:e919523.

Li P, Wang X, Shan Q, Wu Y, Wang Z. 2017. Microrna-130b promotes cell migration and invasion by inhibiting peroxisome proliferator-activated receptor-gamma in human glioma. Oncol Lett. 13(4):2615-2622.

Li P, Xu Y, Wang B, Huang J, Li Q. 2020b. Mir-34a-5p and mir-125b-5p attenuate abeta-induced neurotoxicity through targeting bace1. J Neurol Sci. 413:116793.

Li QS, Cai D. 2021. Integrated mirna-seq and mrna-seq study to identify mirnas associated with alzheimer's disease using post-mortem brain tissue samples. Front Neurosci. 15:620899.

Li W, Zhu Q, Xu X, Hu X. 2021. Mir-27a-3p suppresses cerebral ischemia-reperfusion injury by targeting foxo1. Aging (Albany NY). 13(8):11727-11737.

Li XQ, Lv HW, Wang ZL, Tan WF, Fang B, Ma H. 2015. Mir-27a ameliorates inflammatory damage to the blood-spinal cord barrier after spinal cord ischemia: Reperfusion injury in rats by downregulating ticam-2 of the tlr4 signaling pathway. J Neuroinflammation. 12:25.

Li Z, Chen H. 2019. Mir-34a inhibits proliferation, migration and invasion of paediatric neuroblastoma cells via targeting hnf4alpha. Artif Cells Nanomed Biotechnol. 47(1):3072-3078.

Liang TY, Lou JY. 2016. Increased expression of mir-34a-5p and clinical association in acute ischemic stroke patients and in a rat model. Med Sci Monit. 22:2950-2955.

Liao YC, Wang YS, Guo YC, Lin WL, Chang MH, Juo SH. 2014. Let-7g improves multiple endothelial functions through targeting transforming growth factor-beta and sirt-1 signaling. J Am Coll Cardiol. 63(16):1685-1694.

Liu DZ, Tian Y, Ander BP, Xu H, Stamova BS, Zhan X, Turner RJ, Jickling G, Sharp FR. 2010. Brain and blood microrna expression profiling of ischemic stroke, intracerebral hemorrhage, and kainate seizures. J Cereb Blood Flow Metab. 30(1):92-101.

Liu Y, Ding Y, Hou Y, Yu T, Nie H, Cui Y. 2021. The mir-130a-3p/tgf-betarii axis participates in inhibiting the differentiation of fibroblasts induced by tgf-beta1. Front Pharmacol. 12:732540.

Liu ZD, Wang Q, Pan DQ, Meng FQ, Li JT, Wang YH. 2020. Microrna-130b inhibits cerebral ischemia/reperfusion induced cell apoptosis via regulation of irf1. Eur Rev Med Pharmacol Sci. 24(23):12334-12341.

Lu J, Zhou N, Yang P, Deng L, Liu G. 2019. Microrna-27a-3p downregulation inhibits inflammatory response and hippocampal neuronal cell apoptosis by upregulating mitogen-activated protein kinase 4 (map2k4) expression in epilepsy: In vivo and in vitro studies. Med Sci Monit. 25:8499-8508.

Luo Y, Wu J, Wu Q, Li X, Wu J, Zhang J, Rong X, Rao J, Liao Y, Bin J et al. 2019. Mir-577 regulates tgf-beta induced cancer progression through a sdpr-modulated positive-feedback loop with erk-nf-kappab in gastric cancer. Mol Ther. 27(6):1166-1182.

Lv X, Yan J, Jiang J, Zhou X, Lu Y, Jiang H. 2017. Microrna-27a-3p suppression of peroxisome proliferator-activated receptor-gamma contributes to cognitive impairments resulting from sevoflurane treatment. J Neurochem. 143(3):306-319.

Ma J, Yu J, Liu J, Yang X, Lou M, Liu J, Feng F, Ji P, Wang L. 2017. Microrna-302a targets gab2 to suppress cell proliferation, migration and invasion of glioma. Oncol Rep. 37(2):1159-1167.

Ma ZL, Hou PP, Li YL, Wang DT, Yuan TW, Wei JL, Zhao BT, Lou JT, Zhao XT, Jin Y et al. 2015. Microrna-34a inhibits the proliferation and promotes the apoptosis of non-small cell lung cancer h1299 cell line by targeting tgfbetar2. Tumour Biol. 36(4):2481-2490.

Mao L, Zuo ML, Wang AP, Tian Y, Dong LC, Li TM, Kuang DB, Song GL, Yang ZB. 2020. Low expression of mir5323p contributes to cerebral ischemia/reperfusion oxidative stress injury by directly targeting nox2. Mol Med Rep. 22(3):2415-2423.

Martinelli-Boneschi F, Fenoglio C, Brambilla P, Sorosina M, Giacalone G, Esposito F, Serpente M, Cantoni C, Ridolfi E, Rodegher M et al. 2012. Microrna and mrna expression profile screening in multiple sclerosis patients to unravel novel pathogenic steps and identify potential biomarkers. Neurosci Lett. 508(1):4-8.

Meng Q, Yang P, Lu Y. 2021. Microrna-410 serves as a candidate biomarker in hypoxic-ischemic encephalopathy newborns and provides neuroprotection in oxygen-glucose deprivation-injured pc12 and sh-sy5y cells. Brain Behav. 11(8):e2293.

Mundalil Vasu M, Anitha A, Thanseem I, Suzuki K, Yamada K, Takahashi T, Wakuda T, Iwata K, Tsujii M, Sugiyama T et al. 2014. Serum microrna profiles in children with autism. Mol Autism. 5:40.

Mundy WR, Tilson HA. 1990. Neurotoxic effects of colchicine. Neurotoxicology. 11(3):539-547.

Nakanishi K, Aono S, Hirano K, Kuroda Y, Ida M, Tokita Y, Matsui F, Oohira A. 2006. Identification of neurite outgrowth-promoting domains of neuroglycan c, a brain-specific chondroitin sulfate proteoglycan, and involvement of phosphatidylinositol 3-kinase and protein kinase c signaling pathways in neuritogenesis. J Biol Chem. 281(34):24970-24978.

Ongnok B, Chattipakorn N, Chattipakorn SC. 2020. Doxorubicin and cisplatin induced cognitive impairment: The possible mechanisms and interventions. Exp Neurol. 324:113118.

Organista-Juarez D, Jimenez A, Rocha L, Alonso-Vanegas M, Guevara-Guzman R. 2019. Differential expression of mir-34a, 451, 1260, 1275 and 1298 in the neocortex of patients with mesial temporal lobe epilepsy. Epilepsy Res. 157:106188.

Pan Y, Wang J, He L, Zhang F. 2021. Microrna-34a promotes emt and liver fibrosis in primary biliary cholangitis by regulating tgf-beta1/smad pathway. J Immunol Res. 2021:6890423.

Parchem RJ, Moore N, Fish JL, Parchem JG, Braga TT, Shenoy A, Oldham MC, Rubenstein JL, Schneider RA, Blelloch R. 2015. Mir-302 is required for timing of neural differentiation, neural tube closure, and embryonic viability. Cell Rep. 12(5):760-773.

Piotrzkowska D, Miller E, Kucharska E, Niwald M, Majsterek I. 2021. Association of mirna and mrna levels of the clinical onset of multiple sclerosis patients. Biology (Basel). 10(6).

Poursaei E, Abolghasemi M, Bornehdeli S, Shanehbandi D, Asadi M, Sadeghzadeh M, Rahmanpour D, Sadeh RN. 2021. Evaluation of hsa-let-7d-5p, hsa-let-7g-5p and hsa-mir-15b-5p plasma levels in patients with alzheimer's disease. Psychiatr Genet.

Qiao P, Li G, Bi W, Yang L, Yao L, Wu D. 2015. Microrna-34a inhibits epithelial mesenchymal transition in human cholangiocarcinoma by targeting smad4 through transforming growth factor-beta/smad pathway. BMC Cancer. 15:469.

Rahimmi A, Peluso I, Rajabi A, Hassanzadeh K. 2019. Mir-185 and sept5 genes may contribute to parkinson's disease pathophysiology. Oxid Med Cell Longev. 2019:5019815.

Rao G, Zhang W, Song S. 2019. Microrna217 inhibition relieves cerebral ischemia/reperfusion injury by targeting sirt1. Mol Med Rep. 20(2):1221-1229.

Ravnik-Glavac M, Glavac D. 2020. Circulating rnas as potential biomarkers in amyotrophic lateral sclerosis. Int J Mol Sci. 21(5).

Regis S, Caliendo F, Dondero A, Casu B, Romano F, Loiacono F, Moretta A, Bottino C, Castriconi R. 2017. Tgf-beta1 downregulates the expression of cx3cr1 by inducing mir-27a-5p in primary human nk cells. Front Immunol. 8:868.

Ren Y, Feng J. 2007. Rotenone selectively kills serotonergic neurons through a microtubule-dependent mechanism. J Neurochem. 103(1):303-311.

Robinette BL, Harrill JA, Mundy WR, Shafer TJ. 2011. In vitro assessment of developmental neurotoxicity: Use of microelectrode arrays to measure functional changes in neuronal network ontogeny. Front Neuroeng. 4:1.

Rostamian Delavar M, Baghi M, Safaeinejad Z, Kiani-Esfahani A, Ghaedi K, Nasr-Esfahani MH. 2018. Differential expression of mir-34a, mir-141, and mir-9 in mpp+-treated differentiated pc12 cells as a model of parkinson's disease. Gene. 662:54-65.

Sabirzhanov B, Zhao Z, Stoica BA, Loane DJ, Wu J, Borroto C, Dorsey SG, Faden AI. 2014. Downregulation of mir-23a and mir-27a following experimental traumatic brain injury induces neuronal cell death through activation of proapoptotic bcl-2 proteins. J Neurosci. 34(30):10055-10071.

Sala Frigerio C, Lau P, Salta E, Tournoy J, Bossers K, Vandenberghe R, Wallin A, Bjerke M, Zetterberg H, Blennow K et al. 2013. Reduced expression of hsa-mir-27a-3p in csf of patients with alzheimer disease. Neurology. 81(24):2103-2106.

Salvi A, Vezzoli M, Busatto S, Paolini L, Faranda T, Abeni E, Caracausi M, Antonaros F, Piovesan A, Locatelli C et al. 2019. Analysis of a nanoparticleenriched fraction of plasma reveals mirna candidates for down syndrome pathogenesis. Int J Mol Med. 43(6):2303-2318.

Sanchez-Mora C, Soler Artigas M, Garcia-Martinez I, Pagerols M, Rovira P, Richarte V, Corrales M, Fadeuilhe C, Padilla N, de la Cruz X et al. 2019. Epigenetic signature for attention-deficit/hyperactivity disorder: Identification of mir-26b-5p, mir-185-5p, and mir-191-5p as potential biomarkers in peripheral blood mononuclear cells. Neuropsychopharmacology. 44(5):890-897.

Santos-Bezerra DP, Cavaleiro AM, Santos AS, Suemoto CK, Pasqualucci CA, Jacob-Filho W, Leite REP, Passarelli M, Marie SKN, Machado UF et al. 2021. Alcohol use disorder is associated with upregulation of microrna-34a and microrna-34c in hippocampal postmortem tissue. Alcohol Clin Exp Res. 45(1):64-68.

Shaker FH, El-Derany MO, Wahdan SA, El-Demerdash E, El-Mesallamy HO. 2021. Berberine ameliorates doxorubicin-induced cognitive impairment (chemobrain) in rats. Life Sci. 269:119078.

Shi HZ, Wang DN, Ma LN, Zhu H. 2020a. Microrna-362 inhibits cell growth and metastasis in glioblastoma by targeting mapk1. Eur Rev Med Pharmacol Sci. 24(17):8931-8939.

Shi L, Tian Z, Fu Q, Li H, Zhang L, Tian L, Mi W. 2020b. Mir-217-regulated mef2d-hdac5/nd6 signaling pathway participates in the oxidative stress and inflammatory response after cerebral ischemia. Brain Res. 1739:146835.

Shi W, Du J, Qi Y, Liang G, Wang T, Li S, Xie S, Zeshan B, Xiao Z. 2012. Aberrant expression of serum mirnas in schizophrenia. J Psychiatr Res. 46(2):198-204.

Sun L, Zhao M, Wang Y, Liu A, Lv M, Li Y, Yang X, Wu Z. 2017. Neuroprotective effects of mir-27a against traumatic brain injury via suppressing foxo3a-mediated neuronal autophagy. Biochem Biophys Res Commun. 482(4):1141-1147.

Sun Z, Yu JT, Jiang T, Li MM, Tan L, Zhang Q, Tan L. 2013. Genome-wide microrna profiling of rat hippocampus after status epilepticus induced by amygdala stimulation identifies modulators of neuronal apoptosis. PLoS One. 8(10):e78375.

Supriya M, Christopher R, Indira Devi B, Bhat DI, Shukla D. 2020. Circulating micrornas as potential molecular biomarkers for intracranial aneurysmal rupture. Mol Diagn Ther. 24(3):351-364.

Takousis P, Sadlon A, Schulz J, Wohlers I, Dobricic V, Middleton L, Lill CM, Perneczky R, Bertram L. 2019. Differential expression of micrornas in alzheimer's disease brain, blood, and cerebrospinal fluid. Alzheimers Dement. 15(11):1468-1477.

Tang H, Liu Q, Liu X, Ye F, Xie X, Xie X, Wu M. 2015. Plasma mir-185 as a predictive biomarker for prognosis of malignant glioma. J Cancer Res Ther. 11(3):630-634.

Toma C, Torrico B, Hervas A, Salgado M, Rueda I, Valdes-Mas R, Buitelaar JK, Rommelse N, Franke B, Freitag C et al. 2015. Common and rare variants of microrna genes in autism spectrum disorders. World J Biol Psychiatry. 16(6):376-386.

Toullec D, Pianetti P, Coste H, Bellevergue P, Grand-Perret T, Ajakane M, Baudet V, Boissin P, Boursier E, Loriolle F et al. 1991. The bisindolylmaleimide gf 109203x is a potent and selective inhibitor of protein kinase c. J Biol Chem. 266(24):15771-15781.

Toyama K, Spin JM, Deng AC, Huang TT, Wei K, Wagenhauser MU, Yoshino T, Nguyen H, Mulorz J, Kundu S et al. 2018. Microrna-mediated therapy modulating blood-brain barrier disruption improves vascular cognitive impairment. Arterioscler Thromb Vasc Biol. 38(6):1392-1406.

Tsan YC, Morell MH, O'Shea KS. 2016. Mir-410 controls adult svz neurogenesis by targeting neurogenic genes. Stem Cell Res. 17(2):238-247.

Uhlmann S, Mracsko E, Javidi E, Lamble S, Teixeira A, Hotz-Wagenblatt A, Glatting KH, Veltkamp R. 2017. Genome-wide analysis of the circulating mirnome after cerebral ischemia reveals a reperfusion-induced microrna cluster. Stroke. 48(3):762-769.

van den Berg MMJ, Krauskopf J, Ramaekers JG, Kleinjans JCS, Prickaerts J, Briede JJ. 2020. Circulating micrornas as potential biomarkers for psychiatric and neurodegenerative disorders. Prog Neurobiol. 185:101732.

van Harten AC, Mulders J, Scheltens P, van der Flier WM, Oudejans CB. 2015. Differential expression of microrna in cerebrospinal fluid as a potential novel biomarker for alzheimer's disease. J Alzheimers Dis. 47(1):243-252.

Vandecandelaere A, Martin SR, Engelborghs Y. 1997. Response of microtubules to the addition of colchicine and tubulin-colchicine: Evaluation of models for the interaction of drugs with microtubules. Biochem J. 323 ( Pt 1):189-196.

Wang B, Li Y, You C. 2021a. Mir-129-3p targeting of mcu protects against glucose fluctuation-mediated neuronal damage via a mitochondrial-dependent intrinsic apoptotic pathway. Diabetes Metab Syndr Obes. 14:153-163.

Wang C, Huang S, Rao S, Hu J, Zhang Y, Luo J, Wang H. 2019a. Decreased expression of mir-410-3p correlates with poor prognosis and tumorigenesis in human glioma. Cancer Manag Res. 11:10581-10592.

Wang H, Zhi H, Ma D, Li T. 2017a. Mir-217 promoted the proliferation and invasion of glioblastoma by repressing ywhag. Cytokine. 92:93-102.

Wang J, Fu Z, Wang M, Lu J, Yang H, Lu H. 2021b. Knockdown of xist attenuates cerebral ischemia/reperfusion injury through regulation of mir-362/rock2 axis. Neurochem Res. 46(8):2167-2180.

Wang M, Sun H, Yao Y, Tang X, Wu B. 2019b. Microrna-217/138-5p downregulation inhibits inflammatory response, oxidative stress and the induction of neuronal apoptosis in mpp(+)-induced sh-sy5y cells. Am J Transl Res. 11(10):6619-6631.

Wang Y, Du J, Niu X, Fu N, Wang R, Zhang Y, Zhao S, Sun D, Nan Y. 2017b. Mir-130a-3p attenuates activation and induces apoptosis of hepatic stellate cells in nonalcoholic fibrosing steatohepatitis by directly targeting tgfbr1 and tgfbr2. Cell Death Dis. 8(5):e2792.

Wang Y, Gu J, Hu L, Kong L, Wang T, Di M, Li C, Gui S. 2020. Mir-130a alleviates neuronal apoptosis and changes in expression of bcl-2/bax and caspase-3 in cerebral infarction rats through pten/pi3k/akt signaling pathway. Exp Ther Med. 19(3):2119-2126.

Wang Y, Guan E, Li D, Sun L. 2021c. Mirna-34a-5p regulates progression of neuroblastoma via modulating the wnt/beta-catenin signaling pathway by targeting sox4. Medicine (Baltimore). 100(20):e25827.

Wang Y, Shi M, Hong Z, Kang J, Pan H, Yan C. 2021d. Mir-130a-3p has protective effects in alzheimer's disease via targeting dapk1. Am J Alzheimers Dis Other Demen. 36:15333175211020572.

Wang Y, Wang MD, Xia YP, Gao Y, Zhu YY, Chen SC, Mao L, He QW, Yue ZY, Hu B. 2018. Microrna-130a regulates cerebral ischemia-induced blood-brain barrier permeability by targeting homeobox a5. FASEB J. 32(2):935-944.

Watanabe K, Yamaji R, Ohtsuki T. 2018. Microrna-664a-5p promotes neuronal differentiation of sh-sy5y cells. Genes Cells. 23(3):225-233.

Wei H, Yuan Y, Liu S, Wang C, Yang F, Lu Z, Wang C, Deng H, Zhao J, Shen Y et al. 2015. Detection of circulating mirna levels in schizophrenia. Am J Psychiatry. 172(11):1141-1147.

Wei L, Li P, Zhao C, Wang N, Wei N. 2019. Upregulation of microrna-1270 suppressed human glioblastoma cancer cell proliferation migration and tumorigenesis by acting through wt1. Onco Targets Ther. 12:4839-4848.

Wei N, Wei H, Zhang H. 2018. Long non-coding rna zeb1-as1 promotes glioma cell proliferation, migration and invasion through regulating mir-577. Eur Rev Med Pharmacol Sci. 22(10):3085-3093.

Wu C, Zhang X, Chen P, Ruan X, Liu W, Li Y, Sun C, Hou L, Yin B, Qiang B et al. 2019. Microrna-129 modulates neuronal migration by targeting fmr1 in the developing mouse cortex. Cell Death Dis. 10(4):287.

Wu X, Zhang X, Li D, Zhu Z. 2020. Plasma level of mir-99b may serve as potential diagnostic and short-term prognostic markers in patients with acute cerebral infarction. J Clin Lab Anal. 34(3):e23093.

Xiao QX, Wen S, Zhang XR, Xue LL, Zhang ZB, Tan YX, Du RL, Zhu ZQ, Zhu YH, Wang TH et al. 2020. Mir-410-3p overexpression ameliorates neurological deficits in rats with hypoxic-ischemic brain damage. Brain Res Bull. 162:218-230.

Xiong LL, Xue LL, Du RL, Zhou HL, Tan YX, Ma Z, Jin Y, Zhang ZB, Xu Y, Hu Q et al. 2020. Vi4-mir-185-5p-igfbp3 network protects the brain from neonatal hypoxic ischemic injury via promoting neuron survival and suppressing the cell apoptosis. Front Cell Dev Biol. 8:529544.

Yang IH, Siddique R, Hosmane S, Thakor N, Hoke A. 2009. Compartmentalized microfluidic culture platform to study mechanism of paclitaxel-induced axonal degeneration. Exp Neurol. 218(1):124-128.

Yao L, Guo Y, Wang L, Li G, Qian X, Zhang J, Liu H, Liu G. 2021. Knockdown of mir-130a-3p alleviates spinal cord injury induced neuropathic pain by activating igf-1/igf-1r pathway. J Neuroimmunol. 351:577458.

Yi Z, Shi Y, Zhao P, Xu Y, Pan P. 2020. Overexpression of mir-217-5p protects against oxygen-glucose deprivation/reperfusion-induced neuronal injury via inhibition of pten. Hum Cell. 33(4):1026-1035.

Yin Z, Ma T, Huang B, Lin L, Zhou Y, Yan J, Zou Y, Chen S. 2019. Macrophage-derived exosomal microrna-501-3p promotes progression of pancreatic ductal adenocarcinoma through the tgfbr3-mediated tgf-beta signaling pathway. J Exp Clin Cancer Res. 38(1):310.

Yu HC, Wu J, Zhang HX, Zhang GL, Sui J, Tong WW, Zhang XY, Nie LL, Duan JH, Zhang LR et al. 2015. Alterations of mir-132 are novel diagnostic biomarkers in peripheral blood of schizophrenia patients. Prog Neuropsychopharmacol Biol Psychiatry. 63:23-29.

Zadehbagheri F, Hosseini E, Bagheri-Hosseinabadi Z, Rekabdarkolaee HM, Sadeghi I. 2019. Profiling of mirnas in serum of children with attention-deficit hyperactivity disorder shows significant alterations. J Psychiatr Res. 109:185-192.

Zhang M, Guo Y, Wu J, Chen F, Dai Z, Fan S, Li P, Song T. 2016a. Roles of microrna-99 family in human glioma. Onco Targets Ther. 9:3613-3619.

Zhang R, Zhang Q, Niu J, Lu K, Xie B, Cui D, Xu S. 2014. Screening of micrornas associated with alzheimer's disease using oxidative stress cell model and different strains of senescence accelerated mice. J Neurol Sci. 338(1-2):57-64.

Zhang W, Shen C, Li C, Yang G, Liu H, Chen X, Zhu D, Zou H, Zhen Y, Zhang D et al. 2016b. Mir-577 inhibits glioblastoma tumor growth via the wnt signaling pathway. Mol Carcinog. 55(5):575-585.

Zhang Y, Chen M, Qiu Z, Hu K, McGee W, Chen X, Liu J, Zhu L, Wu JY. 2016c. Mir-130a regulates neurite outgrowth and dendritic spine density by targeting mecp2. Protein Cell. 7(7):489-500.

Zhang Y, Wu Z, Li X, Wan Y, Zhang Y, Zhao P. 2020. Maternal sevoflurane exposure affects differentiation of hippocampal neural stem cells by regulating mir-410-3p and atn1. Stem Cell Res Ther. 11(1):423.

Zheng Y, Wang L, Chen M, Pei A, Xie L, Zhu S. 2017. Upregulation of mir-130b protects against cerebral ischemic injury by targeting water channel protein aquaporin 4 (aqp4). Am J Transl Res. 9(7):3452-3461.

Zhong C, Tao B, Yang F, Xia K, Yang X, Chen L, Peng T, Xia X, Li X, Peng L. 2021. Histone demethylase jmjd1c promotes the polarization of m1 macrophages to prevent glioma by upregulating mir-302a. Clin Transl Med. 11(9):e424.

Zhou J, Liu J, Pan Z, Du X, Li X, Ma B, Yao W, Li Q, Liu H. 2015. The let-7g microrna promotes follicular granulosa cell apoptosis by targeting transforming growth factor-beta type 1 receptor. Mol Cell Endocrinol. 409:103-112.

Zhu C, Huang L, Xu F, Li P, Li P, Hu F. 2020a. Lncrna pcat6 promotes tumor progression in osteosarcoma via activation of tgf-beta pathway by sponging mir-185-5p. Biochem Biophys Res Commun. 521(2):463-470.

Zhu X, Liu X, Liu Y, Chang W, Song Y, Zhu S. 2020b. Uncovering the potential differentially expressed mirnas and mrnas in ischemic stroke based on integrated analysis in the gene expression omnibus database. Eur Neurol. 83(4):404-414.

Zhu Y, Zhao H, Feng L, Xu S. 2016. Microrna-217 inhibits cell proliferation and invasion by targeting runx2 in human glioma. Am J Transl Res. 8(3):1482-1491.

Zou D, Zhou X, Liu J, Zhao Y, Jiang X. 2020. Mir-34a regulates schwann cell proliferation and migration by targeting cntn2. Neuroreport. 31(17):1180-1188.

**Supplemental Figures**

**Note that supplemental figures are also included as separate image files*

**Supplemental Figure 1. Ingenuity Pathway Analysis Workflow for miRNAs.** The flowchart describes the steps taken for filtering, network construction, and functional analyses to identify miRNA-mRNA associations, related pathways, and enrich for the notable miRNAs.


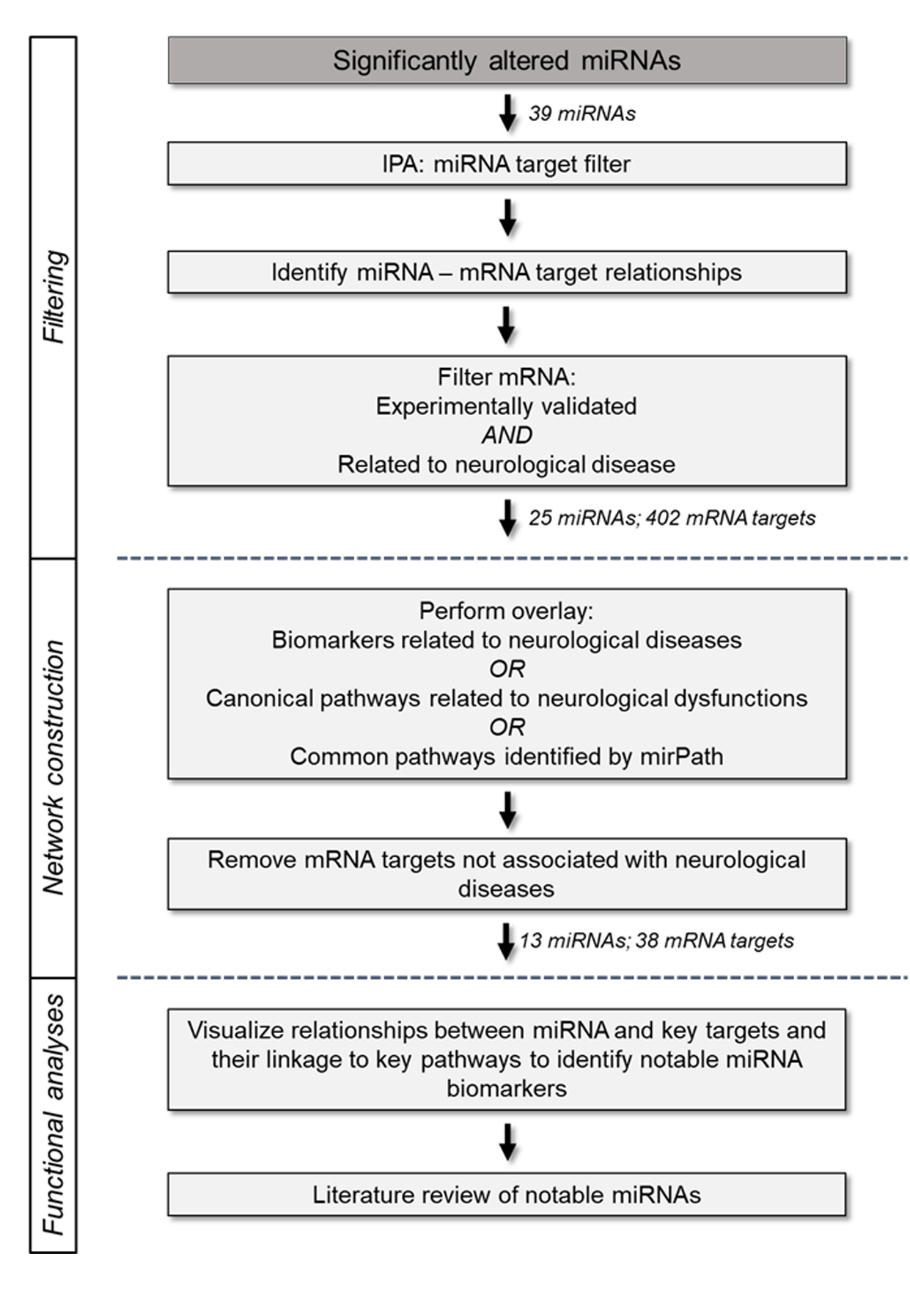


**Supplemental Figure 2. Representative images of human iPSC-neurons after 24-hours chemical treatment.** Images were taken at 20x magnification, scale bar = 100 μm, and green = βIII-tubulin (neurons) and blue = DAPI (nuclei). Images adapted from Cohen and Tanaka 2018.


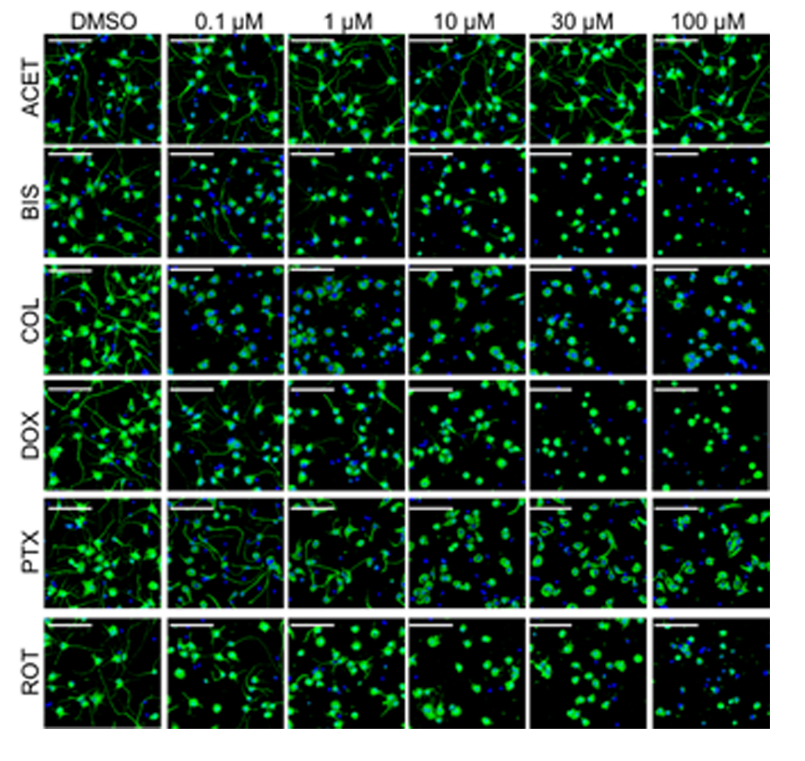

Supplement: kfac011_Supplementary_Data [file kfac011_supplementary_data.zip › kfac011-suppl_data/toxsci-21-0524-File010.docx]
